# Transcriptional modulation unique to vulnerable motor neurons predicts ALS across species and SOD1 mutations

**DOI:** 10.1101/gr.279501.124

**Published:** 2025-09

**Authors:** Irene Mei, Susanne Nichterwitz, Melanie Leboeuf, Jik Nijssen, Isadora Lenoel, Dirk Repsilber, Christian S. Lobsiger, Eva Hedlund

**Affiliations:** 1 Department of Biochemistry and Biophysics, Stockholm University, 106 91 Stockholm, Sweden;; 2 Department of Neuroscience, Karolinska Institutet, 171 77 Stockholm, Sweden;; 3 Department of Cellular and Molecular Biology, Karolinska Institutet, 171 77 Stockholm, Sweden;; 4 Sorbonne Université, Institut du Cerveau - Paris Brain Institute - ICM, Inserm, CNRS, APHP, Hôpital de la Pitié Salpêtrière, 75013 Paris, France;; 5 School of Medical Sciences, Örebro University, 701 82 Örebro, Sweden

## Abstract

Amyotrophic lateral sclerosis (ALS) is characterized by the progressive loss of motor neurons (MNs) that innervate skeletal muscles. However, certain MN groups including ocular MNs, are relatively resilient. To reveal key drivers of resilience versus vulnerability in ALS, we investigate the transcriptional dynamics of four distinct MN populations in SOD1G93A ALS mice using LCM-seq and single-molecule fluorescent in situ hybridization. We find that resilient ocular MNs regulate few genes in response to disease. Instead, they exhibit high baseline gene expression of neuroprotective factors, including *En1*, *Pvalb*, *Cd63*, and *Gal*, some of which vulnerable MNs upregulate during disease. Vulnerable MN groups upregulate both detrimental and regenerative responses to ALS and share pathway activation, indicating that breakdown occurs through similar mechanisms across vulnerable neurons, albeit with distinct timing. Meta-analysis across four rodent mutant *Sod1* MN transcriptome data sets identify a shared vulnerability code of 39 genes, including *Atf4*, *Nupr1*, *Ddit3*, and *Penk*, involved in apoptosis, as well as a proregenerative and antiapoptotic signature consisting of *Atf3*, *Vgf*, *Ina*, *Sprr1a*, *Fgf21*, *Gap43*, *Adcyap1*, and *Mt1*. Machine learning using genes upregulated in SOD1G93A spinal MN predicts disease in human stem cell–derived *SOD1E100G* MNs and shows that dysregulation of *VGF*, *INA*, and *PENK* is a strong disease predictor across species and *SOD1* mutations. Our study reveals MN population-specific gene expression and temporal disease-induced regulation that together provide a basis to explain ALS selective vulnerability and resilience and that can be used to predict disease.

Amyotrophic lateral sclerosis (ALS) is a neurodegenerative disease that is characterized by the loss of upper motor neurons (MNs) in the cortex, leading to spasticity, and lower somatic MNs in the brainstem and spinal cord that control skeletal muscles, leading to muscle atrophy and paralysis. About 10% of cases are familial, and of these, ∼20% are caused by dominant mutations in the superoxide dismutase 1 (*SOD1*) gene, which induces ALS through gain-of-toxic function mechanisms (Rosen et al. 1993). *SOD1* is ubiquitously expressed in the body, and when mutated, it accumulates across cell types over time. Mutant *SOD1* affects the function of many cell types, and yet, it causes selective loss of particular neurons only. In fact, not even all somatic MNs are affected in ALS, but certain subpopulations show a strong resilience, including oculomotor (cranial nerve [CN] 3), trochlear (CN4) and abducens (CN6) MNs, which innervate the extraocular muscles that control eye movement. Their resilience has been demonstrated in sporadic ALS patients (Gizzi et al. 1992; Kubota et al. 2000; Caligari et al. 2013), in mutant SOD1 ALS mice (Tjust et al. 2012; Valdez et al. 2012; Comley et al. 2016), and in inducible mutant TARDBP (also known as TDP-43) mice (Spiller et al. 2016). Thus, this resilience is present across disease causations and species. Furthermore, visceral MNs that innervate smooth muscle appear relatively unaffected in ALS patients (Shimizu et al. 2011; McLeod et al. 2022). Why particular neurons are more resilient than others to ALS and if this is entirely intrinsically programmed or also owing to differences in the environments surrounding MNs or in their muscle targets are still unclear. By removing mutant *SOD1* from specific cell types in transgenic mice, studies have demonstrated that mutant SOD1 within MNs is crucial for initiating and driving early disease progression (Boillée et al. 2006). In contrast, microglia and astrocytes play a more prominent role during later stages of disease progression (Boillée et al. 2006; Yamanaka et al. 2008). Mouse embryonic stem cell (mESC)–derived CN3/4 neurons also show a higher level of resistance to ALS-like toxicity in vitro, indicating that part of their resilience comes from within (Allodi et al. 2019). Furthermore, CN3/4 MNs express relatively high levels of different growth factors at baseline and in response to disease, which can protect MNs and their synapses with muscle (Allodi et al. 2016; Nichterwitz et al. 2020). CN3/4 MNs also lack expression of *Mmp9*, which is highly expressed in vulnerable spinal MNs and, when knocked out, can render these more resistant (Kaplan et al. 2014). Thus, the presence of certain factors and the absence of others, combined with the neuronal response to disease, determine cellular resilience. Analysis of the transcriptional dysregulation in vulnerable spinal MNs in SOD1-ALS mice (Perrin et al. 2005; Lobsiger et al. 2007; Sun et al. 2015; Shadrach et al. 2021) has revealed a response to DNA damage and cell injury as well as activation of compensatory regeneration.

So far no comprehensive analysis has been conducted to dissect how vulnerable versus resilient neurons respond to ALS over time. We reasoned that an analysis of how differentially vulnerable neurons respond to mutant *SOD1* over time, as well as careful consideration of their baseline gene expression, could give further insight into disease mechanisms in ALS. We analyzed different types of resilient MNs, including somatic CN3/4 MNs, and visceral MNs of the dorsal motor vagus nerve (CN10) to delineate if resilient neurons respond in a similar fashion to ALS. We included two vulnerable MN subpopulations, lumbar spinal MNs and hypoglossal MNs (CN12), to also answer the question if vulnerable neurons share destructive pathways and an attempt of compensatory responses or if the brainstem and spinal cord are going through distinct disease mechanisms, owing to differences in target innervation and local environments. We used laser capture microdissection (LCM) and Smart-seq2 RNA sequencing (LCM-seq) (Nichterwitz et al. 2016, 2018) to analyze the transcriptional dynamics across these neuronal populations in ALS mice and wild-type littermates and confirmed differential gene expression (DEG) using RNAscope. We also conducted machine learning, using the random forest classifier and Lasso regression, to identify strong disease predictors across the *SOD1* mutations and species, as well as a meta-analysis on existing transcriptomic data sets of rodent mutant *Sod1* spinal MNs to reveal a general vulnerability code.

## Results

### Transcriptional regulation of mutant *Sod1* or other disease-associated genes does not underlie selective vulnerability among MNs

**Figure 1.**
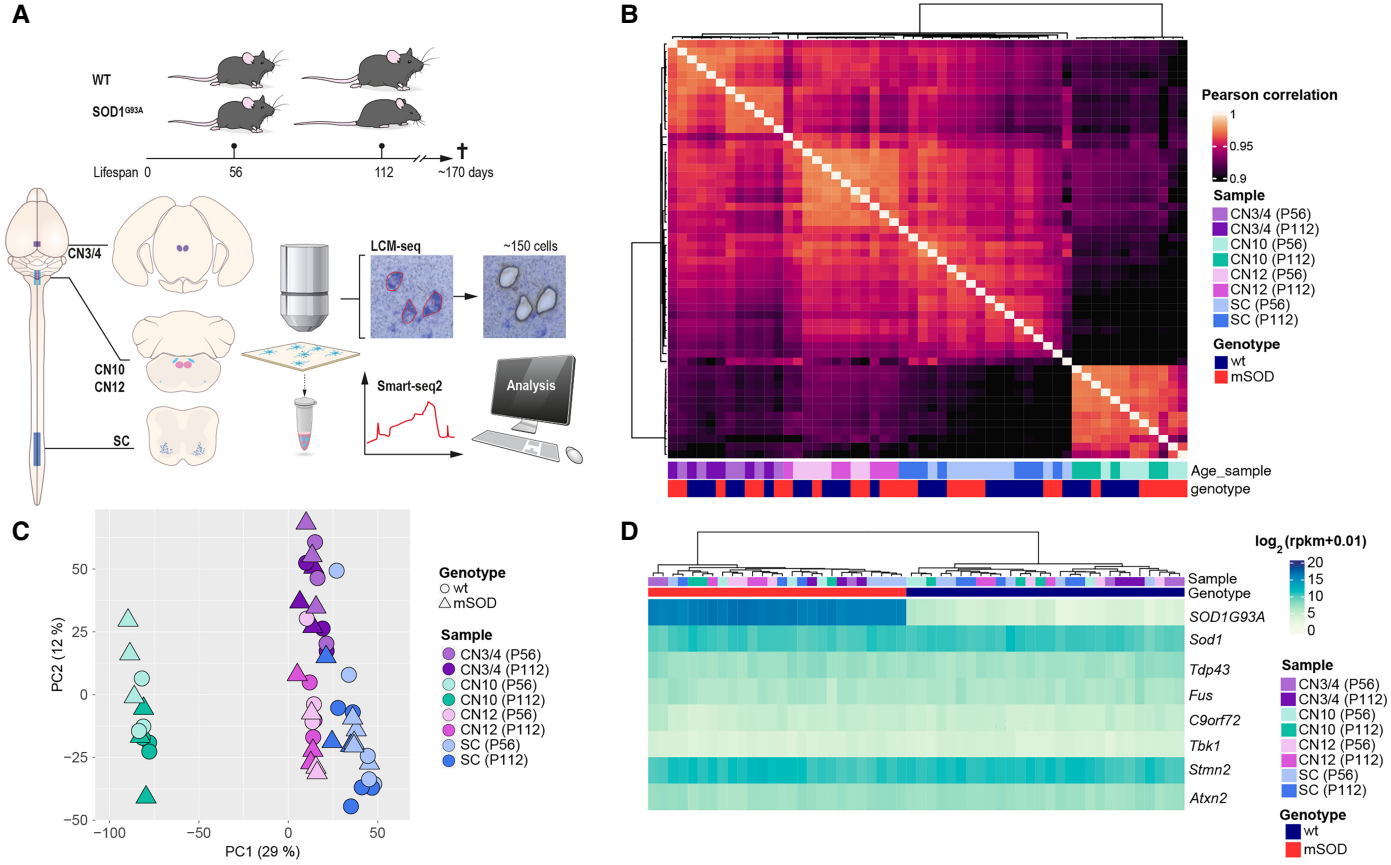
Motor neuron (MN) subpopulations show unique transcriptional profiles but similar levels of ALS disease gene expression. (*A*) Schematic representation of the study design and laser capture microdissection (LCM) coupled with RNA sequencing (LCM-seq) workflow. We used SOD1G93A mice, a well-established ALS model, and their wild-type (WT) littermates as controls at postnatal day 56 (P56; presymptomatic) and postnatal day 112 (P112; onset of symptoms). LCM was performed to isolate MNs from three cranial nerve (CN) nuclei (CN3/4, CN10, CN12) and the lumbar (L5) spinal cord (SC), followed by Smart-seq2 RNA sequencing for transcriptome analysis. (*B*) Pairwise Pearson's correlation heatmap of variance-stabilized transformed (VST) gene expression data, showing hierarchical clustering of samples by cell type, genotype, and age. A single linkage method was used for the hierarchical clustering of columns. (*C*) Principal component analysis (PCA) based on whole-transcriptome expression data highlights clear separation between different MN subtypes, with genotype and age also influencing clustering. (*D*) Heatmap of key ALS-associated genes (*Sod1*, *Tardp43*, *Fus*, *C9orf72*, *Tbk1*, *Stmn2*, *Atxn2*), showing no major differences across MN populations. (CN12) Hypoglossal nucleus, (CN10) dorsal motor nucleus of the vagus nerve, (CN3/4) oculomotor and trochlear nuclei.

To retrieve temporal mechanistic insight into the differential vulnerability across MN populations in ALS, we isolated somatic MNs from CN3/4 (resilient), CN12 (vulnerable), and lumbar spinal cord (highly vulnerable), as well as visceral MNs from CN10 (resilient) of presymptomatic (P56) and onset-of-symptoms (P112) SOD1G93A mice and wild-type littermates using LCM. We subjected the neurons to Smart-seq2 poly(A)-based RNA sequencing (Fig. 1A; Supplemental Fig. S1). Reads uniquely mapped to the mouse genome (69.7 ± 0.50% mean ± SEM) were used for further analyses (Supplemental Fig. S2A). Samples clustered by cell type with a Pearson's correlation of at least 0.95 and with no separation between ALS and controls (Fig. 1B; see Supplemental Methods).

**Figure 2.**
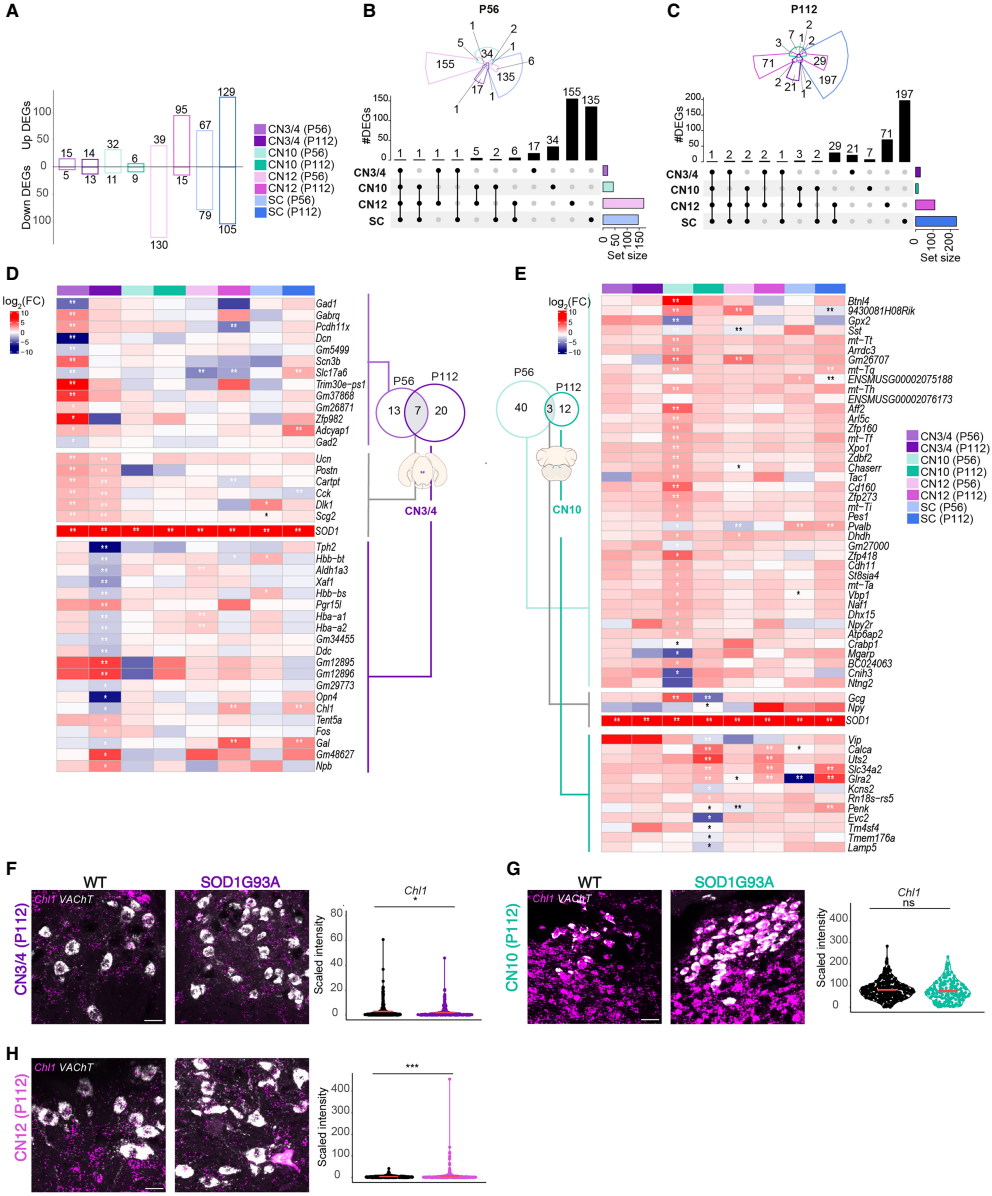
Relatively resistant MNs show little gene dysregulation in response to mutant SOD1. (*A*) Bar plots of differentially expressed genes (DEGs) across MN populations in SOD1G93A versus WT mice, highlighting a low number of DEGs in CN3/4 compared with other MN populations. Numbers on *top* and *bottom* of each bar represent, respectively, the number of up and down DEGs. (*B*,*C*) Upset plots of DEGs at P56 and P112, showing the number of genes differentially expressed across CN3/4, CN10, CN12, and SC at different disease stages. Likelihood ratio test, Benjamini–Hochberg adjusted *P-*value < 0.05. (*D*) Heatmap showing log fold change (logFC) values of the DEGs across disease stages for CN3/4 at both P56 and P112, showing relatively few significant changes compared with other MN populations. Likelihood ratio test, (**) Benjamini–Hochberg adjusted *P*-value < 0.01, (*) Benjamini–Hochberg adjusted *P*-value < 0.05. (*E*) Heatmaps showing log expression of the DEGs across disease stages for CN10. Likelihood ratio test, (**) Benjamini–Hochberg adjusted *P*-value < 0.01, (*) Benjamini–Hochberg adjusted *P*-value < 0.05. (*F*–*H*) RNAscope images of *Chl1* mRNA expression in CN3/4 (n for WT = 376; n for SOD1G93A = 319), CN10 (n for WT = 377; n for SOD1G93A = 206), and CN12 (n for WT = 530; n for SOD1G93A = 508), at P112 with quantification of signal intensity. Scale bars, 30 µm. Permutation test (ns) *P* > 0.05, (*) *P* ≤ 0.05, (**) *P* ≤ 0.01, (***) *P* ≤ 0.001, (****) *P* ≤ 0.0001.

We conducted hierarchical clustering of samples based on *Phox2a/b* and *Hox* gene expression, as these transcription factors distinctly define MN populations along the anterior–posterior (A-P) body axis of vertebrates (Pattyn et al. 2000; Dasen et al. 2005). This confirms that CN3/4 neurons cluster based on *Phox2b* expression and lack of *Hox* gene expression, whereas spinal MNs cluster based on caudal *Hox* gene expression (*Hox6-11*) compared with CN10 and CN12 neurons, as expected (Supplemental Fig. S2B; Allodi et al. 2019; Nichterwitz et al. 2020). Further marker gene analysis showed that all MN groups expressed *Chat*, *Isl1* (also known as *Islet 1*), peripherin (*Prph*), and neurofilament, heavy polypeptide (*Nefh*). CN12 and spinal MNs also expressed *Isl2* and *Mnx1*, whereas those markers were nearly absent in CN3/4 and visceral CN10 MNs (Supplemental Fig. S2C). Principal component analysis (PCA) including all expressed genes showed that samples cluster based on cell type (Fig. 1C). PC1 clearly distinguished visceral (CN10) from somatic (CN3/4, CN12, and spinal) MNs. The PC1 also, to a lesser extent, distinguished brainstem MNs from spinal MNs, whereas PC2 distinguished CN3/4 MNs from the other groups (Fig. 1C). ALS neurons were not separated from wild-type neurons (Fig. 1C), as only a minority of genes were dysregulated in ALS compared with the overall gene expression across these neurons (Fig. 2A). Analysis of additional PCs (PC3–6) did not further resolve diseases versus control (Supplemental Fig. S2D,E). The ALS mouse model is based on overexpression of human mutated *SOD1G93A*, and the level of the resulting protein is important for disease development. Analysis of human and mouse *Sod1* levels shows that human *SOD1* mRNA is highly expressed in SOD1G93A mice across neuron types and that endogenous mouse *Sod1* mRNA is expressed across all mice, as anticipated (Fig. 1D). We conducted a careful statistical analysis of mutant *SOD1* mRNA levels across cell types. Human *SOD1* expression varied only slightly across cell types, with CN10 and CN12 MNs showing the highest levels and no significant changes across disease stage (Supplemental Fig. S2F). We conducted an ANOVA comparing the two *Sod1* gene homologs, followed by Bonferroni-corrected post-hoc *t*-test, which revealed only very subtle differences between the groups. Collectively, these small differences in mRNA level of *SOD1G93A* do not reflect the vulnerability of the respective cell types. Thus, differential regulation of transcripts other than *SOD1G93A* and *Sod1* hold the key to understanding differential vulnerability in SOD-ALS. Analysis of mESC-derived cranial MNs has indicated that these have higher proteasome activity compared with spinal MNs and thus may be more resilient to proteostatic stress and misfolded SOD1 (An et al. 2019). To investigate this matter in vivo, we analyzed GO terms related to proteasome, ubiquitination, and proteolysis across samples of CN3/4 and spinal MNs, using gene set variation analysis (GSVA) (Hänzelmann et al. 2013). We found no indication at the transcriptome level that CN3/4 MNs would have a better capacity to degrade proteins than spinal MNs in wild-type or SOD1G93A mice (Supplemental Fig. S2G), but this does not exclude translational or post-translational regulation of proteasome activity or differences in mESC-derived versus adult mouse MNs. We subsequently analyzed the expression levels of other disease-causing or disease-modifying genes, including *Tardbp*, *Fus*, *C9orf72*, *Tbk1*, *Stmn2*, and *Atxn2*, but we found no differences across cell types or disease states (Fig. 1D). In conclusion, different MN types are clearly distinguished by their transcriptomic profiles, and *SOD1G93A*-induced gene expression changes do not shift the identity of the MNs in any major way. *SOD1G93A* expression levels were comparable across neuron types and thus do not reflect differences in susceptibility, neither does the expression level of other investigated disease-causative or -modifying genes. Therefore, to understand selective vulnerability, further analysis of genes and pathways dysregulated downstream of mutant *SOD1G93A* may reveal how cells respond differently and thus either succumb to or are shielded from disease.

### MN subpopulations show unique spatial and temporal gene regulation in response to mutant *Sod1* expression

To retrieve mechanistic insight into the selective vulnerability and resilience of particular MN groups in ALS, we analyzed differential gene expression induced by mutant *SOD1G93A* overexpression (Supplemental Tables S1–S9). We found that resilient groups, CN3/4 and CN10, regulate very few genes with disease compared with the vulnerable CN12 and spinal MNs (Fig. 2A).

Analysis at the presymptomatic P56 stage demonstrated that each MN population responds largely uniquely to mSOD1 (Fig. 2B; Supplemental Fig. S3A). At the P112 onset of symptoms stage, most DEGs were still unique to the particular MN group. Resilient CN3/4 and CN10 neurons only shared *mSOD1* as a DEG and thus clearly do not show a common induced-resilience signature to ALS. However, the vulnerable CN12 and spinal MNs showed a larger overlap in DEGs at the onset of symptoms than at the presymptomatic stage, with an overlap of 34 genes in total (Fig. 2C; Supplemental Fig. S3A).

### Upregulation of a few genes with known neuroprotective properties is sufficient for CN3/4 MNs to cope with disease

To understand the spatially restricted temporal gene expression changes in disease, we analyzed the DEGs within each motor nucleus across the P56 and P112 time points. Starting with the resilient MNs, we identified 13 DEGs unique to CN3/4 at the presymptomatic stage that recovered to baseline at the symptom-onset time, for example, gamma-aminobutyric acid type A receptor subunit theta (*Gabrq*) (Fig. 2D; Supplemental Fig. S3B). Notably, *Gabrq* levels were pronouncedly higher in CN3/4 and CN10 than in vulnerable MN groups, which may regulate their excitability (Supplemental Fig. S3B). In CN3/4, seven genes were dysregulated across disease stages, including urocortin (*Ucn*) (Pedersen et al. 2002), cholecystokinin (*Cck*) (Wozniak et al. 2021), and the ECM glycoprotein periostin, osteoblast specific factor (*Postn*) (Shimamura et al. 2012), which all have known neuroprotective properties. *Postn* was not only upregulated in CN3/4 MNs with disease (Fig. 2D) but also showed several-fold higher expression levels in CN3/4 MNs than all other groups at baseline in control mice (Supplemental Fig. S3C). CART prepropeptide (*Cartpt*), delta like non-canonical Notch ligand 1 (*Dlk1*), secretogranin II (*Scg2*) (Fig. 2D), and *SOD1G93A* were the remaining shared CN3/4 DEGs. Twenty genes were uniquely regulated in CN3/4 MNs at the symptom-onset stage. Notably, *Chl1*, which was reduced in CN3/4, showed the opposite regulation in CN12 and spinal MNs (Fig. 2D). For CN10, 43 DEGs were identified at P56 and 15 DEGs at P112, with no overlap with CN3/4 in terms of gene regulation (Fig. 2E).

We used RNAscope fluorescence in situ hybridization to validate gene expression changes. A probe detecting *Slc18a3* (also known as *VAChT*) was used to identify MNs. The signal was systematically quantified in each MN identified from the *VAChT* channel using an automated CellProfiler pipeline (v.4.2.5) (Supplemental Fig. S4A; see Supplemental Methods; Stirling et al. 2021), with the number of neurons analyzed per gene probe shown in Supplemental Figure S4B.

The downregulation of *Chl1* at P112 in CN3/4 MNs identified through LCM-seq was confirmed (*P* ≤ 0.05) (Fig. 2F), as was the lack of regulation in CN10 MNs (Fig. 2G) and the upregulation in CN12 MNs at the same time point (*P* ≤ 0.001) (Fig. 2H).

To identify if any cellular pathways were dysregulated with disease in resilient MNs, we conducted a comprehensive analysis employing three distinct pathway analysis methods: overlap-based analysis (OVA) utilizing the EASE method, per-gene analysis (PGA) employing FGSEA, and network enrichment analysis (NEA) with the ANUBIX method. No pathways were significantly changed in CN3/4 MNs with disease, whereas in CN10 MNs, four pathways were dysregulated at P112 (Supplemental Fig. S3D; Supplemental Tables S10–S18). In conclusion, based on the DEGs identified in resilient neuron groups, it is evident that there is no common induced-resilience signature across neuron types. Some of the DEGs identified, including *Ucn*, *Cck*, and *Postn*, point to neuroprotective pathways that may explain some of the tolerance toward mutant *SOD1G93A*. Nonetheless, with so few transcriptional changes identified in resilient neurons, it appears conceivable that part of their resilience is encoded in their baseline gene expression.

### Identification of an innate resilience code of CN3/4 MNs and its partial induction in vulnerable MNs with SOD1-ALS

**Figure 3.**
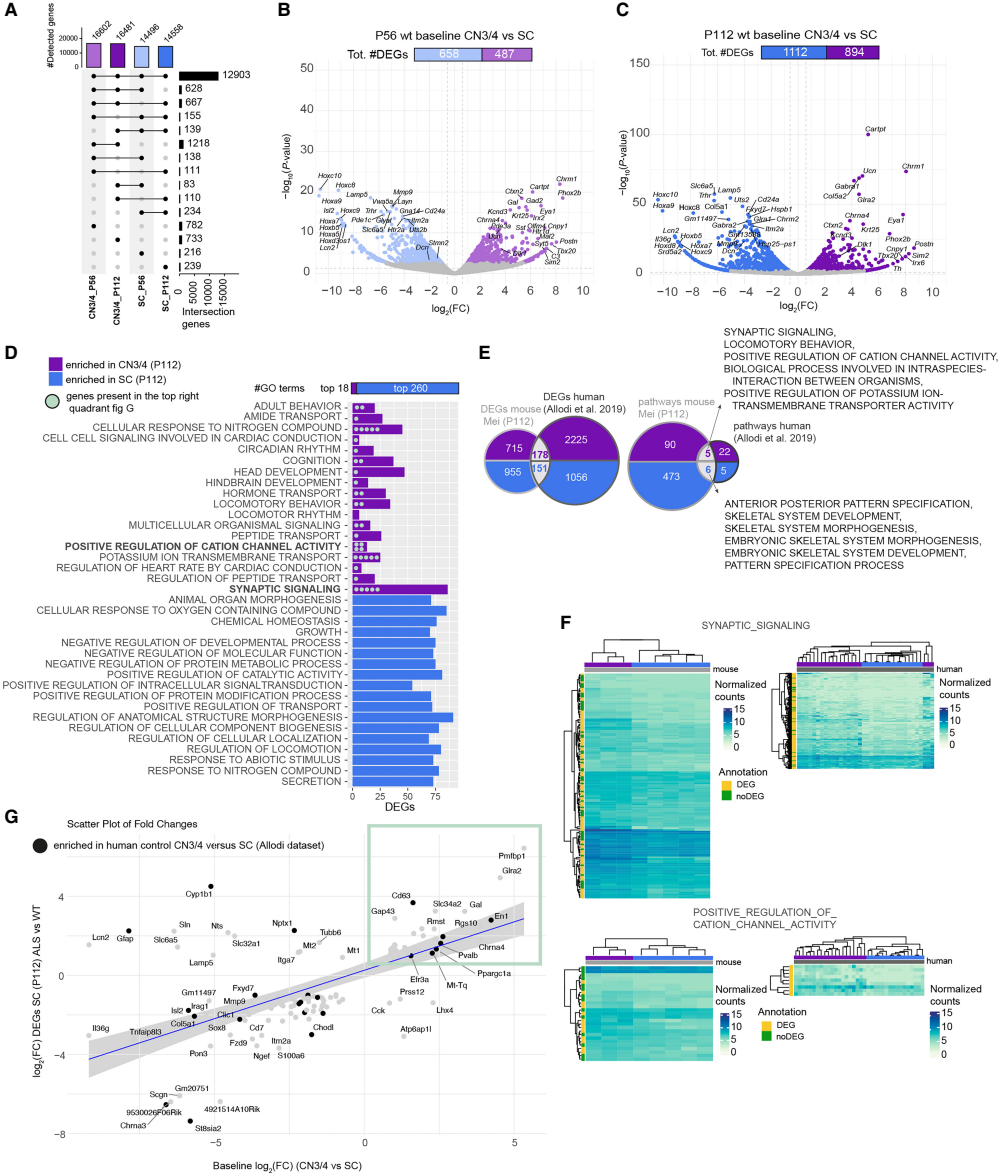
Baseline gene expression in CN3/4 MNs may hold the key to their resilience. (*A*) Upset plot showing the number of detected genes in CN3/4 and SC in WT mice at P56 and P112. (*B*) Volcano plot of DEGs at P56 comparing CN3/4 (light purple) versus SC (light blue), highlighting genes enriched in each population. Likelihood ratio test, Benjamini–Hochberg adjusted *P-*value < 0.05. (*C*) Volcano plot of DEGs at P112 comparing CN3/4 (purple) versus SC (blue), showing an increased number of DEGs at the symptom-onset stage. Likelihood ratio test, Benjamin–Hochberg adjusted *P-*value < 0.05. (*D*) Gene Ontology (GO) term enrichment analysis performed using FGSEA showing enriched GO terms in WT CN3/4 or WT spinal (SC) MNs at P112. (*E*) Comparison of DEGs and enriched pathways between the mice (Mei_P112 data set) and humans (Allodi et al. 2019). (*F*) Heatmaps of selected pathways and genes enriched in control CN3/4 MNs in mice and in humans. (*G*) Scatter plot comparing ALS-induced expression changes in SC (P112) with baseline gene expression differences between CN3/4 and SC, identifying genes that may contribute to CN3/4 resistance. Each dot corresponds to a gene. Genes labeled in bold represent those also identified in the human baseline data set (Allodi et al. 2019). The blue line represents the linear regression of fold changes, with the shaded region showing the 95% confidence interval. Highlighted genes in the green box exhibit higher expression in CN3/4 baseline and are induced in our SC at P112 samples, making them potential candidates for further investigation.

The combination of few gene regulation changes occurring in resilient neurons and of no pathways identified in CN3/4 MNs in response to *mSOD1*, prompted us to investigate the general basal gene expression signature unique to CN3/4 MNs in control mice. We reasoned that this may explain why resilient neurons do not need to modulate their transcriptomes to a large extent in response to toxic gene expression. We found that the vast majority of transcripts were shared across resilient CN3/4 and vulnerable spinal MNs, as expected. CN3/4 MNs had 1218 uniquely detected transcripts shared across the two time points and an additional 782 transcripts unique to P56 and 733 genes unique to P112, which we reasoned could underlie their relative resilience to ALS (Fig. 3A). The transcripts enriched in either MN population were visualized using Volcano plots (Fig. 3B,C; Supplemental Tables S19, S20). Spinal MNs were significantly enriched in, for example, *Hox8-10* family members, *Mmp9*, *Dcn*, *Trhr*, and *Uts2*, whereas CN3/4 MNs were enriched in, for example, *Phox2b*, *Tbx20*, *Eya1*, *Gabra1*, *Gabrb2*, *Glra2*, *Mt-Tq*, *Dlk1*, *Chrm1*, and *Ucn* (Fig. 3B,C; Supplemental Figs. S3E,F, S5). This cell type–specific marker enrichment is in line with previously published studies (Hedlund et al. 2010; Kaplan et al. 2014; Nichterwitz et al. 2020).

We subsequently analyzed GO terms enriched in either MN group at P112 and found that 18 GO terms were enriched in CN3/4 neurons (normalized enrichment score [NES] > 0), whereas 260 were enriched in spinal MNs (NES < 0) (Fig. 3D; Supplemental Tables S21, S22). To further define CN3/4-enriched gene expression valid across species, we compared several published transcriptomics data sets: Allodi et al. (2019) (obtained from the NCBI Gene Expression Omnibus [GEO; https://www.ncbi.nlm.nih.gov/geo/] under accession number GSE93939; LCM-seq of human control CN3/4 and spinal MNs), Brockington et al. (2013) (GSE40438; microarray of human control CN3/4 and spinal MNs), and Kaplan et al. (2014) (GSE52118; microarray of P9 control mouse CN3/4 and spinal MNs). The initial analysis showed that only 17 DEGs were shared across all data sets (Supplemental Fig. S6A,B). RNA-seq data sets revealed a substantially larger number of DEGs between CN3/4 and spinal MNs compared with microarray analyses (Supplemental Fig. S6A), and we thus focused on the RNA-seq data. Our cross-comparison with Allodi et al. (2019) on human postmortem control CN3/4 and spinal MNs identified 178 CN3/4-enriched DEGs and 151 spinal MN-enriched DEGs across species (Fig. 3E). Pathway analysis revealed five shared CN3/4-enriched GO terms, including “synaptic signaling” and “positive regulation of cation channel activity” (Fig. 3F) and six enriched in spinal MNs across species, including, for example, “anterior-posterior pattern specification” and “skeletal system development” (Fig. 3E). To dissect the neuroprotective program innate to CN3/4 MNs, we analyzed which transcripts enriched in CN3/4 MNs at baseline were induced in vulnerable MNs with ALS, the reasoning being that vulnerable neurons induce transcript for protection when in distress. This analysis highlighted that vulnerable spinal MNs upregulate a set of CN3/4-enriched transcripts in response to the SOD1 mutation, including *En1*, *Gal*, *Pvalb*, *Gap43*, *Glra2*, *Cd63*, *Rgs10*, *Mt-Tq*, and *Rmst* (highlighted in Fig. 3G, green box). We also found that several of these transcripts, which are considered neuroprotective, were enriched in CN3/4 MNs in human control postmortem tissues, including *EN1*, *CD63, PVALB*, and *MT-TQ* (highlighted additionally with black points in Fig. 3G, green box).

In conclusion, our complementary analysis of the baseline gene expression in CN3/4 versus spinal MNs highlights their fundamental differences, on top of which gene expression shifts occur with disease, mainly in vulnerable neurons. Our analysis clearly points out that basal cell type–specific gene expression needs to be considered in addition to differential gene expression with disease. In particular, our analysis highlights a set of genes and pathways that are enriched in CN3/4 MNs at baseline (without disease), which are in part induced in vulnerable spinal MNs in response to SOD1-ALS. These are presumed to contribute to a protective response, which is maintained at a high level in resilient neurons without an apparent need for further regulation there, and are insufficiently induced in vulnerable MNs in an attempt to keep them at bay in ALS.

### Vulnerable MNs show unique regulation of injury response genes indicative of cellular stress, tissue remodeling, and MN subtype switching

**Figure 4.**
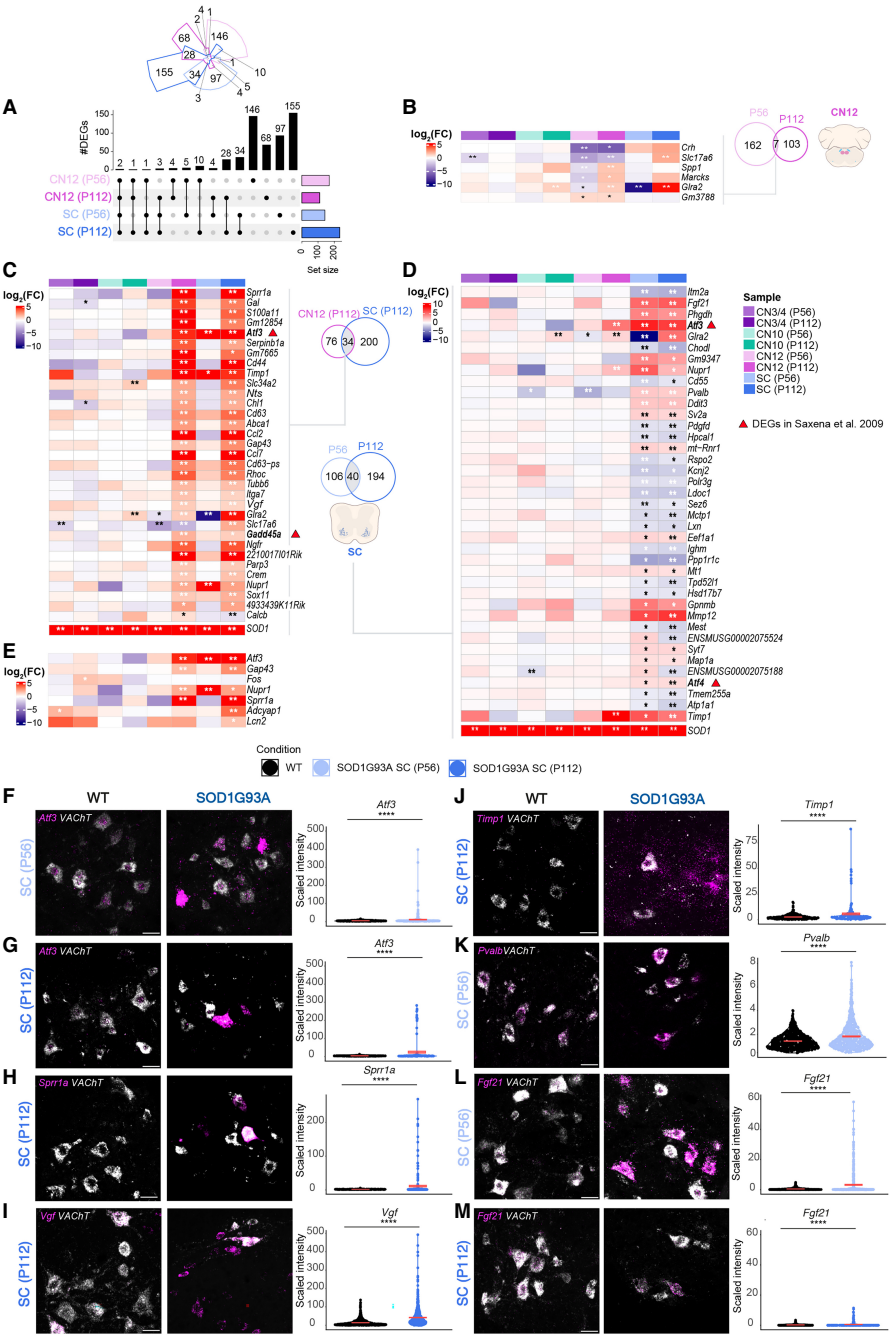
Vulnerable MNs show unique regulation of injury response genes. (*A*) Upset plot showing DEGs in vulnerable populations (CN12 and SC) at P56 and P112. Likelihood ratio test, Benjamini–Hochberg adjusted *P-*value < 0.05. (*B*) Heatmap showing logFC expression of shared DEGs between ages in CN12. (*C*) Heatmap showing logFC expression of common DEGs between CN12 and SC at P112. (*D*) Heatmap showing logFC expression of common DEGs between ages in SC MNs. (*E*) Heatmap showing logFC expression of DEGs related to nerve injury response. (*B–E*) Genes in bold represent DEGs also dysregulated in the work of Saxena et al. (2009). Likelihood ratio test, (**) Benjamini–Hochberg FDR < 0.01, (*) Benjamini–Hochberg FDR < 0.05. Representative RNAscope images with quantification of signal intensity of *Atf3* in SC at P56 (n for WT = 407; n for SOD1G93A = 281; *F*) and P112 (n for WT = 340; n for SOD1G93A = 136; *G*), *Sprr1a* in SC at P112 (n for WT = 321; n for SOD1G93A = 218; *H*), *Vgf* in SC at P112 (n for WT = 1031; n for SOD1G93A = 704; *I*), *Timp1* in SC at P112 (n for WT = 340; n for SOD1G93A = 136; *J*), *Pvalb* in SC at P56 (n for WT = 606; n for SOD1G93A = 523; *K*), and representative RNAscope image of *Fgf21* in SC at P56 (n for WT = 592; n for SOD1G93A = 508; *L*) and P112 (n for WT = 434; n for SOD1G93A = 258; *M*). (*F*–*M*) Scale bars, 30 µm. Permutation test, (ns) *P* > 0.05, (*) *P* ≤ 0.05, (**) *P* ≤ 0.01, (***) *P* ≤ 0.001, (****) *P* ≤ 0.0001.

We next set out to fully dissect how relatively vulnerable MN subpopulations respond to disease and the extent of temporal and spatial overlap in gene regulation. Our initial analysis clearly demonstrates that gene dysregulation in vulnerable neurons is tightly regulated in both time and space. Globally, CN12 and spinal MNs showed a majority of transcriptional responses that were unique to their individual cell types and disease states (Fig. 4A). However, in contrast to the lack of overlap between the two resilient MN populations (Fig. 2B,C), there was significant overlap between the two vulnerable MN populations at the later disease stage (112 days) (Fig. 4A). Thus, to comprehensively understand how disease progresses, it is necessary to conduct longitudinal analysis as it is difficult to predict the steps that will follow. Furthermore, one cell type does not necessarily inform about another, pressing the point that it is pivotal to study disease across cell types. Nonetheless, we reasoned that some of the shared responses across time may give deep insight into continuous disease predictors and drivers.

For CN12, only seven of 169 DEGs from P56 were DEGs also at P112; however, only half of these genes were regulated in the same direction with time (Fig. 4A,B). Of these, *Glra2* and particularly *Slc17a6*, which has neuroprotective capacities (Steinkellner et al. 2018), were also induced in spinal MNs at symptom onset and may be a protective response to damage. P112 CN12 MNs showed a higher overlap in gene dysregulation with same age spinal MNs compared with P56 CN12 MNs, indicative of a shared, later-stage disease response across vulnerable MNs. To investigate this general vulnerability code, we analyzed the 34 DEGs shared at P112 (Fig. 4C). We identified several genes uniquely upregulated with disease across vulnerable MNs, including *Atf3*, *Cd44*, *Gadd45a*, *Ngfr*, *Ccl2*, *Ccl7*, *Gal*, *Timp1*, *Nupr1*, *Serpinb1a*, *Ch1*, *Vgf*, *Sprr1a*, and *Gap43* (Fig. 4C,D; Supplemental Fig. S3G, *Vgf*). For spinal MNs, 40 DEGs were shared across the time points, whereas 106 DEGs were unique to the presymptomatic P56 time point and 194 DEGs to the symptom-onset P112 stage, including *Nupr1*, *Atf4*, *Ddit3*, *En1*, *Gap43*, *Chl1*, *Cd44*, *Rhoc, Tubb6*, *Timp1*, *Mmp9*, *Glra2*, *Gabrg2*, *Penk*, *Chrna4*, and *Dlg4* (Supplemental Tables S8, S9; Supplemental Fig. S3H, *Penk*). Some of the 40 DEGs shared across time points were uniquely upregulated in spinal MNs, including *Fgf21*, *Mt1*, *Sv2a*, *Chodl*, *Gpnmb*, *Mmp12*, *Syt7*, *Map1a*, *Atf4*, *Atp1a1*, *Ddit3*, and *Pvalb* (Fig. 4D). *Chodl*, which is a known marker of FF MNs (Enjin et al. 2010), was consistently downregulated with disease in spinal MNs, whereas *Sv2a*, a marker of slow MNs, was upregulated (Fig. 4D), suggesting ongoing compensatory processes across MN subtypes early on in disease. On the other hand, transcripts belonging to ongoing programmed neuronal death were only regulated in the later stages in spinal MNs (only at P112) and were not seen in CN12, including *Adcyap1* and *Lcn2* (Fig. 4D,E), suggesting that P56 spinal MNs are not yet at an advanced stage of neuronal damage and neither are CN12 MNs at P112.

The upregulation of *Atf3* in vulnerable MNs (Fig. 4C) was confirmed by RNAscope at both the presymptomatic stage (*P* ≤ 0.00001) (Fig. 4F) and at the symptom-onset stage (*P* ≤ 0.00001) (Fig. 4G). *Atf3* was also upregulated at presymptomatic stage in vulnerable CN12 MNs (*P* ≤ 0.0172) (Supplemental Fig. S7B,D). Although the symptom-onset time point was not significant, some neurons were completely filled with *Atf3* mRNA, which was never seen in the control (Supplemental Fig. S7D). *Atf3* was not upregulated in CN3/4 MNs at either time point (Supplemental Fig. S7A,C), in concordance with the LCM-seq data. Consistent with axonal sprouting occurring alongside axonal degeneration, *Gap43* expression was increased in both vulnerable populations at the symptom-onset stage (Fig. 4C–E). *Sprr1a* was also found upregulated across vulnerable MNs, concordant with a response to axon damage in ALS (Fig. 4C–E), and RNAscope on spinal cord sections confirmed the upregulation with disease at the symptom-onset stage (*P* ≤ 0.0001) (Fig. 4H). In CN3/4 MNs *Sprr1a* was undetectable, as expected (Supplemental Fig. S7E), whereas it was strongly induced in some CN12 MNs at P112, confirming the LCM-seq result (*P* ≤ 0.0001) (Supplemental Fig. S7F).

*Vgf* was induced in both vulnerable populations at P112, and this upregulation was confirmed by RNAscope in spinal MNs (*P* ≤ 0.00001) (Fig. 4I). We also confirmed the upregulation of *Timp1* in spinal (*P* ≤ 0.0001) (Fig. 4J), and CN12 (*P* ≤ 0.0001) (Supplemental Fig. S7H) MNs at P112, whereas it remained unchanged in CN3/4 MNs (Supplemental Fig. S7G), consistent with the LCM-seq data and indicative of ongoing tissue remodeling.

*Pvalb*, which was upregulated in spinal MNs, similar to what we have previously seen at the protein level in the SOD1G93A mouse (Comley et al. 2015), and downregulated in CN12 MNs with disease (Fig. 4D), was also confirmed by RNAscope at the presymptomatic stage (Fig. 4K, *P* ≤ 0.00001; Supplemental Fig. S7J, *P* < 0.0001). *Pvalb* was also slightly downregulated in CN3/4 MNs with disease at the presymptomatic stage (*P* < 0.0001) (Supplemental Fig. S7I), following the pattern of regulation seen in the LCM-seq data (Fig. 4D). *Fgf21* was confirmed upregulated in spinal MNs at P56 (*P* ≤ 0.00001) (Fig. 4L) and P112 (*P* = 7 × 10^−7^) (Fig. 4M). *Fgf21* was also slightly upregulated in both CN3/4 and CN12 MNs at P56 according to the RNAscope analysis (*P* = 0.0187 for CN3/4 and *P* < 0.0001 for CN12) (Supplemental Fig. S7K,L). In the LCM-seq data, this difference was not significant but showed a trend toward an increase (Fig 4D). Thus, all the in situ data from six different probes were concordant with our RNA-seq analysis, showing regulation in SOD1G93A MNs.

### Vulnerable MN subpopulations degenerate in a similar fashion but at distinct temporal paces

**Figure 5.**
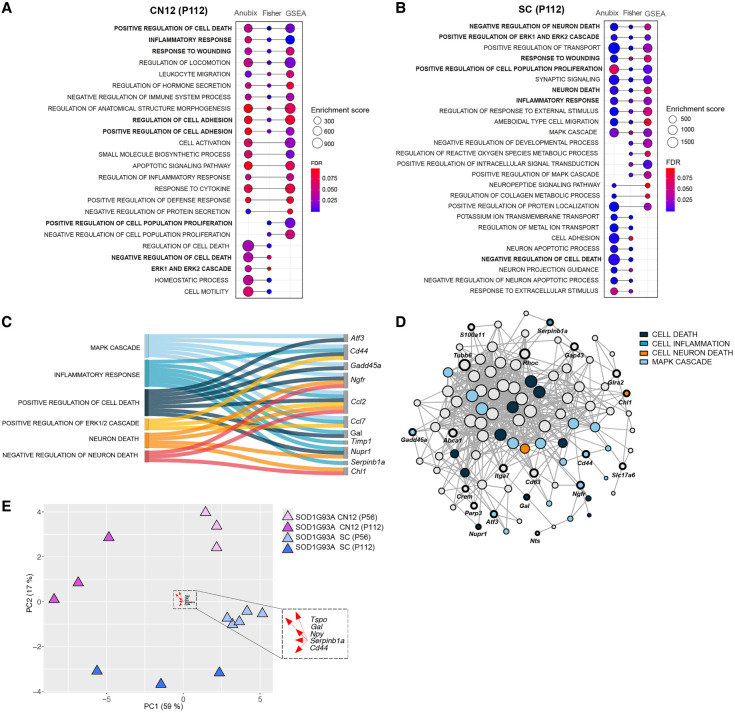
Pathway analysis shows a common detrimental response across vulnerable populations. (*A*,*B*) GO enrichment dot plots showing significantly enriched pathways that were enriched in at least two of the pathway analysis methods (Fisher test, FGSEA, Anubix) for CN12 and SC at P112. Enrichment scores correspond to the number of functional genes that the method shows being related for the enrichment term. FDR threshold <0.1. (*C*) Sankey plot showing functional categorization of stress-related genes in CN12 and SC at P112. (*D*) Functional network analysis on genes shared between CN12 and SC at P112 using Funcoup 5 with all evidence types. Evidence types are the signals that support or contradict the presence of functional coupling. In Funcoup 5, the evidence types included are domain interactions, genetic interaction profile similarity, gene regulation, mRNA coexpression, microRNA regulation, protein coexpression, phylogenetic profile similarity, physical interaction, subcellular localization, and transcription factor binding profile. (*E*) PCA plot illustrates the clustering pattern of samples based on DEGs belonging to common detrimental pathways (“positive regulation of cell death,” “inflammatory response,” “response to wounding,” “negative regulation of cell death,” “ERK1 and ERK2 cascade”) for CN12 and SC cell types at P112. The top five loading genes for PC1 are highlighted in the plot.

To elucidate the pathways activated in vulnerable MNs, we conducted a comprehensive enrichment analysis (EA) employing methods from three different categories: OVA, PGA, and NEA. We selected EASE (OVA) (Hosack et al. 2003), FGSEA (PGA) (Korotkevich et al. 2021), and ANUBIX (NEA) (Castresana-Aguirre and Sonnhammer 2020), being representative of their categories. Pathways showing enrichment were identified using an FDR cutoff of 0.1. The pathways demonstrating the most robust enrichment, and thus consensus across the three methods, included, for example, “regulation of neuronal death,” “inflammatory response,” “regulation of ERK cascade,” “MAPK cascade,” “regulation of cell adhesion,” “cell migration,” and “synaptic signaling” (Fig. 5A,B). Further analysis demonstrated that six of the major affected pathways were driven by 11 genes (*Atf3*, *Cd44*, *Gadd45a*, *Ngfr*, *Ccl2*, *Ccl7*, *Gal*, *Timp1*, *Nupr1*, *Serpinb1a*, and *Chl1*) (Fig. 5C), which were part of the 34 DEGs commonly induced between SC and CN12 (Fig 4C). To investigate commonality in gene network activation across vulnerable neurons, we analyzed the DEGs shared in spinal and CN12 MNs at P112 using Funcoup 5 (Alexeyenko and Sonnhammer 2009; https://funcoup.org/search/). This analysis demonstrated connectivity between *Gadd45*, *Ngfr*, *Atf3*, and *Cd44* in the MAPK cascade and integration with *Chl1* in the “cell neuron death pathway,” *Nupr1* in the “cell death pathway,” and *Serpinb1a* in the “cell inflammation pathway” (Fig. 5D). Moreover, using the DEGs related to common detrimental pathways in CN12 and SC at P112, we saw a clear temporal divergence along PC1 in the PCA plot (Fig. 5E).

Our results so far reveal that there is a stress gene signature unique to vulnerable neurons. This response is to a large extent shared across different vulnerable MNs, but the timing is distinct, with CN12 MNs showing a later onset and response to disease compared with spinal MNs. This result is similar to what Saxena et al. (2009) demonstrated for S versus FF MNs, in which the less vulnerable S MNs showed a similar response to the more vulnerable FF, but later in the disease process. Notably, their data were generated using microarrays rather than RNA-seq. Several of the markers identified there, including *Atf3*, *Atf4*, and *Gadd45a*, overlap with our screen on vulnerable MNs (Fig. 4C,D). The lower overlap of DEGs in CN12 over time compared with spinal MNs likely reflects that these neurons are not as advanced in the disease process. As a result, detrimental as well as regenerative processes are not activated at the presymptomatic stage, but only at the symptom onset. In contrast, these processes are activated across all disease stages in spinal MNs. This analysis solidifies our finding that CN12 MNs are not as far along in the disease process as spinal MNs but are progressing on a similar path.

### Machine learning and meta-analysis across SOD1 mutations and models identify strong disease predictors

**Figure 6.**
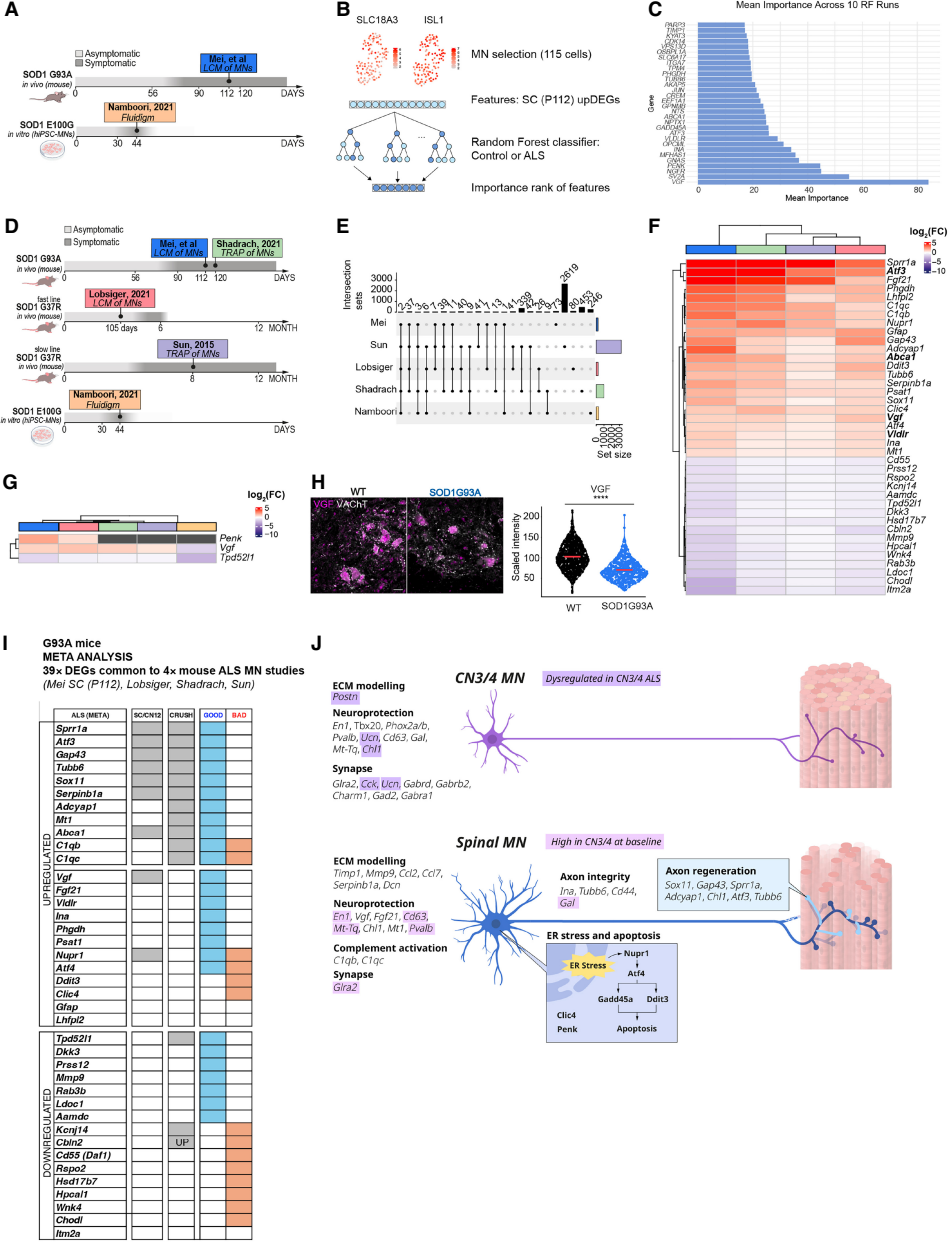
Data set comparison and feature selection show a common importance for *Vgf* in ALS. (*A*) Overview of the comparison between two data sets: the Namboori et al. (2021) (human) and SC (P112; mouse) data sets. (*B*) Schematic of the random forest–based machine learning approach for identifying key ALS-relevant genes using upregulated DEGs in SC (P112; n cells selected in Namboori et al. data set: 115). (*C*) Feature importance ranking from the random forest classifier, highlighting *VGF* as the most predictive marker. (*D*) Overview of cross-data set DEG comparison, including work of Shadrach et al. (2021), Lobsiger et al. (2007), Sun et al. (2015), and Namboori et al. (2021). (*E*) Upset plot showing DEGs shared across ALS data sets. Likelihood ratio test, Benjamini–Hochberg adjusted *P-*value < 0.05. (*F*) Heatmap of the 39 common DEGs identified across all mouse studies between SC at P112, Shadrach et al. (2021), Sun et al. (2015), and Lobsiger et al. (2007). Bolded gene names are also predictive in the random forest disease classifier. (*G*) Heatmap showing common DEGs between all data sets. Colors represent log_2_(FC); black squares indicate that that gene did not result as a DEG for the relative data set. (*H*) Representative immunofluorescence image of SC at P112 and showing the expression of VGF protein in VAChT-positive cells. Scale bar, 30 µm. Welch's two-sample *t*-test *P* ≤ 2.2 × 10^−16^. (*I*) Heatmap displays the 39 ALS-induced DEGs (*F*) common to four mutant *SOD1* MN studies (Mei, Lobsiger, Shadrach, and Sun). Genes are categorized as upregulated or downregulated in ALS MNs. The presence of each gene in different data sets is indicated in columns MEI (disease-induced DEGs shared between CN12 and SC MNs at P112 from Mei et al.) and CRUSH (sciatic nerve crush model from Shadrach et al. 2021), as well as if the regulation is considered “GOOD” or “BAD.” (*J*) Summary of gene expression and regulation in CN3/4 and spinal (SC) MNs in healthy animals and SOD1-ALS. (*Top*) CN3/4-enriched genes are involved in ECM remodeling, neuroprotection, and synapse function, with those regulated in ALS highlighted in purple. (*Bottom*) Gene expression dysregulated in spinal MNs in SOD1-ALS is involved in ECM remodeling, neuroprotection, complement activation, synapse function, axon integrity, ER stress and apoptosis, and axon regeneration. Genes normally enriched in healthy CN3/4 MNs compared with spinal MNs that are induced in spinal MNs with ALS include the neuroprotective genes *En1*, *Fgf21*, *Mt-Tq*, and *Pvalb* and ECM-related genes.

Next, we set out to reveal which of the 129 upregulated DEGs identified in spinal SOD1G93A MNs at P112 (Fig. 2A) would be the strongest disease predictors. Toward this purpose, we used an independent single-cell RNA-seq data set, Namboori et al. (2021), on induced pluripotent stem cell (iPSC)–derived neurons harboring a *SOD1E100G* mutation (or corrected control iPSCs), at a time when the MNs were starting to degenerate in culture (Fig. 6A). From these RNA-seq data, we selected MNs (N = 115) only, based on their coexpression of *SLC18A3* and *ISL1*. We used a random forest classifier (Breiman 2001) as our machine learning approach to evaluate if our DEGs could classify these MNs into ALS or control (Fig. 6B). Our classification model achieved an average sensitivity of 73.3% (*P* < 0.002), specificity of 72.9% (*P* < 0.002), PPV of 48.9% (*P* < 0.002), NPV of 88.6% (*P* < 0.002), AUC of 84.9% (*P* < 0.002), and accuracy of 73.0% (*P* < 0.002). The top genes contributing to the classification, based on permutation-based importance, were *VGF*, *SV2A*, *PENK*, neurotensin (*NTS*), and internexin neuronal intermediate filament protein alpha (*INA*) (Fig. 6C). For assessing disease predictors (diagnostic markers) with an alternative approach, we also applied a Lasso regression model (Tibshirani 1996), which yielded an AUC of 72% (*P* < 0.002). Notably, *VGF*, *SV2A*, and *PENK* were consistently identified among the top predictive features (Supplemental Fig. 8A,B).

We next evaluated if these disease-predicting DEGs were regulated in spinal MNs in response to mutant *SOD1* across data sets. We reasoned that shared regulation across mouse cohorts, mutations, time points, and even species would reveal important disease regulators. Such a meta-analysis across models and *SOD1* mutations had not been done previously and is clearly challenging, as there is a temporal regulation in gene expression with disease, as seen in our SOD1G93A data. Nonetheless, we identified overlap across time points, particularly in the vulnerable MN groups, speaking to the feasibility of our approach. We thus analyzed three additional published data sets that isolated RNA from spinal MNs from mice overexpressing human mutant *SOD1*, as well as the Namboori et al. sequencing data on human iPSC-derived MNs: (1) Lobsiger et al. (2007) isolated MNs from lumbar spinal cord from onset of symptoms (15 weeks of age) in a fast-progressing SOD1G37R mouse (reaches end-stage at 6 months) using LCM and Affymetrix arrays; (2) Sun et al. (2015) used TRAP to isolate mRNAs on ribosomes in MNs at symptom onset (8 months of age) in a slow-progressing SOD1G37R mouse (reaches end-stage at 12 months); and (3) Shadrach et al. (2021) used TRAP to isolate RNA on ribosomes in MNs in symptomatic SOD1G93A (4 months of age) (Fig. 6D). The four mouse studies, including the present one, shared 39 DEGs (Fig. 6E,F; Supplemental Tables S23–S25). Among these were *Vgf*, *Gap43*, *Ina*, *Psat1*, *Clic4*, and *Abca1* (Fig. 6F), which all aided in the classification of human MNs into ALS or control (Fig. 6C). *Penk*, which was a strong disease predictor in the human data set (Fig. 6C) was identified in our data and that of Lobsiger et al. (2007; Fig. 6G). Only two DEGs were found across all five studies (mouse and human), and those were *Vgf* and *Tpd52l1* (Fig. 6G). Although only upregulated DEGs were used to classify the human data set (Fig. 6C), *Tpd52l1* was consistently downregulated across data sets and thus may also predict disease (Fig. 6F). *Vgf* was also upregulated in CN12 with disease but remained unaffected in resilient MNs (Fig. 4C). Furthermore, it should be noted that although *Vgf* was upregulated in all four mouse studies, it was downregulated in the work of Namboori et al. (2021; Fig. 6G). A previous study showed that the VGF protein level in cerebrospinal fluid (CSF) was decreased in ALS patients by ∼40%, and VGF immunoreactivity in postmortem spinal MNs was also decreased (Zhao et al. 2008). As we saw the opposite pattern on the mRNA level across all mouse studies, we decided to analyze the VGF protein level using immunofluorescence staining of SOD1G93A spinal cords and wild-type littermates (N = 5/genotype). Our analysis showed that VGF protein was indeed decreased in MNs with disease (*P* ≤ 2.2 × 10^−16^) (Fig. 6H), consistent with previous findings in patients (Zhao et al. 2008). Thus, the mRNA increase of *Vgf* may be a compensatory event owing to the loss of VGF protein. To get a comprehensive understanding of common DEGs and their implications for disease, we compared them with RNA-seq data of MNs after nerve crush (using TRAP to isolate RNA on ribosomes in MNs) (Shadrach et al. 2021), which would delineate regenerative versus detrimental responses (Fig. 6I). We clustered gene regulation as either “GOOD” or “BAD” responses for ALS. This analysis clearly indicates that ALS MNs do not simply degenerate, but there are protective compensatory processes activated along with the deleterious. Overall “GOOD” responses included neuroprotective genes that were upregulated: *Sprr1a*, *Atf3*, and *Gap43* mediating proregenerative responses; *Phgdh* and *Psat1* (L-serine pathway) acting neurotrophic; and *Adcyap1* and *Mt1* as protective responses against stress that were upregulated. It also included downregulation of detrimental genes including *Mmp9* (vulnerability marker) and *Ldoc1* (proapoptosis). In contrast, overall detrimental (BAD) responses included upregulation of proapoptosis genes *Clic4* and *Nupr1*, and downregulation of neuroprotective genes *Cbln2*, *Daf1* (complement inhibitor), *Cbln2* (synapse formation), and *Rspo2* (NMJ formation) (Fig. 6I). Finally, to further interpret and summarize our findings, both on differences found in baseline gene expression between resilient and vulnerable neurons as well as on their responses to disease, we investigated functional categories, which revealed key pathways linked to ER stress and apoptosis, axon integrity and regeneration, neuroprotection, synapse, complement activation, and ECM remodeling (Fig. 6J). In conclusion, we have identified a resilience code in ALS as well as strong ALS disease markers and predictors based on DEGs identified in our study and across multiple published data sets, which predict disease across SOD1 mutations and species.

## Discussion

We conducted RNA sequencing analysis of MNs displaying differential vulnerability to degeneration in ALS to unveil mechanisms of protection and degeneration at distinct disease stages. Our analysis did not reveal any major differences with respect to mutant *SOD1* mRNA levels across neuron types or in other disease-associated gene expression that could explain their differences in vulnerability. Thus, the cause for selective susceptibility to ALS lies elsewhere, and analysis of responses induced by mutant *SOD1* may give mechanistic insight into why certain neurons succumb to disease and why others persist.

Analysis of differential gene expression consistently showed that resilient MNs display a very mild transcriptomic response to SOD1-ALS, whereas vulnerable MNs showed large transcriptional dysregulation already early in disease. This is different from the gene dysregulation we previously noted in spinal muscular atrophy (SMA), in which resilient CN3/4 MNs elicited a large early gene response compared with vulnerable spinal and facial (CN7) MNs, which only responded to the loss of the *Smn1* gene (survival MN 1) later in disease. In the SMA mouse model, we saw an induction of a large set of intuitively protective genes in CN3/4 MNs in combination with a disease module that was shared across vulnerable and resilient neurons (Nichterwitz et al. 2020). This indicates that CN3/4 MNs are not as severely affected by misfolded mutant SOD1 as they are to loss of SMN and consequent disruption of RNA splicing. It is also consistent with CN3/4 MNs showing less aggregation of misfolded SOD1 compared with vulnerable MNs, as demonstrated in SOD1G93A (An et al. 2019) and in SOD1G85R (Thomas et al. 2018) mice.

Even though SOD-ALS resilient neurons, both CN3/4 and CN10, regulated very few genes and thus did not seem in need to modulate gene expression in a major way to survive, the gene expression changes that did occur may hold a partial key to their resilience. Seven genes were dysregulated across disease stages in CN3/4 MNs: *Ucn*, *Postn*, *Cartpt*, *Cck*, *Dlk1*, *Scg2*, and *mSod1*. Of these DEGs, *Ucn* has been shown to protect hippocampal neurons from oxidative stress and excitotoxic glutamate insult at picomolar levels (Pedersen et al. 2002). *Cck* has been shown to protect Purkinje cells from toxicity in ataxias (SCA mice) (Wozniak et al. 2021). *Postn* levels not only were upregulated in CN3/4 MNs with disease but also were several-fold higher in CN3/4 MNs than all other groups at baseline in control mice. POSTN is an ECM glycoprotein that can stimulate neurite outgrowth (Shih et al. 2014) and be neuroprotective (Shimamura et al. 2012). To comprehend why CN3/4 MNs do not need to regulate a large number of genes to cope with disease, we conducted a complementary analysis of the baseline expression differences between CN3/4 and spinal cord MNs, which highlighted the fundamental differences between these MNs. Here it became evident that although CN3/4 MNs expressed a somewhat larger number of genes, the number of GO terms uniquely enriched in each nucleus was 14 times larger in spinal MNs with 260 enriched GO terms compared with only 18 in CN3/4 MNs. Thus, CN3/4 MNs may require fewer processes for their function than spinal MNs do, maybe because of their lower metabolic demand and output onto far fewer muscle fibers.

Our cross-comparison with neurons isolated from human postmortem tissues identified a number of pathways unique to each MN group that was conserved across species, including the CN3/4-enriched GO terms of “synaptic signaling” and “positive regulation of cation channel activity.” To further visualize the innate neuroprotective program of CN3/4 MNs, we analyzed which CN3/4-enriched transcripts were induced in vulnerable MNs with ALS, with the hypothesis that vulnerable neurons induce transcripts for protection when in distress. Our analysis highlights that several CN3/4-enriched baseline transcripts, including *En1*, *Pvalb*, *Gap43*, *Glra2*, *Gal*, *Cd63*, *Rgs10*, and *Rmst*, are induced in vulnerable MNs with SOD1-ALS. Of these transcripts, *EN1*, *CD63*, *PVALB*, and *MT-TQ* were also relatively enriched in CN3/4 MNs compared with spinal MNs in postmortem tissues from control subjects. This indicates that baseline gene expression differences that may explain CN3/4 MN resilience is shared across species and highlights *En1* as a potential CN3/4-resilience factor. In fact, EN1 is known to act as a neuroprotective factor to MNs. In the spinal cord, EN1 is normally not produced by MNs, but rather by V1 spinal interneurons, that synapse on alpha MNs and deliver this homeodomain protein paracrine. If this delivery is blocked, MNs start to degenerate (Leboeuf et al. 2023). Our finding that *En1* mRNA is also induced within vulnerable MNs in ALS clearly demonstrates an attempt at neuroprotection, similar to what CN3/4 MNs display already at baseline without being challenged and maintain throughout disease.

Our analysis also pinpoints that to understand vulnerability and resilience, we need to examine baseline gene expression difference in connection with disease-induced dysregulation.

In addition to DEG analysis, we also made a first attempt to examine polyadenylation in our data. To this end, we found that both CN3/4 and spinal MNs show large changes in polyadenylation with disease, a response that was partially overlapping across populations and time, but also show unique regulation related to the particular MN group in question and the disease stage (Supplemental Fig. S9A–H). This opens up a new interesting avenue for future research using complementary approaches to investigate the fate of particular modified transcripts to elucidate if this leads to changes in subcellular localization of transcripts, their splicing, and/or half-life of the resulting proteins.

Several of the genes regulated in vulnerable spinal MNs were previously shown to be upregulated in spinal MNs in mutant SOD1 mice (Lobsiger et al. 2007). Our study reveals that this gene signature is unique to vulnerable neurons and shared across SOD1 mutations and is part of a stress response to disease. Vulnerable spinal MNs showed an upregulation of a gene set known to be important for nerve regeneration, including *Gap43*, *Cd44*, *Chl1*, *Atf3*, *Sprr1a*, and *Adcyap1*. This highlights that vulnerable MNs during ALS are actively trying to overcome the ongoing degeneration by inducing genes that stimulate regeneration. It has also been shown that certain spinal MNs are able to sprout and compensate for the muscle denervation of neighboring MNs. This results in a temporary stabilization of NMJ loss versus gain, which is thought to slow the decline in motor dysfunction (Frey et al. 2000; Fischer et al. 2004; Schaefer et al. 2005; Comley et al. 2016). Reduction of *Cd44* has been shown to reduce axon initiation of retinal ganglion cells (Ries et al. 2007), and thus, an increase as seen in the SOD1G93A MNs is expected to promote axon growth. The upregulation of *Atf3* in vulnerable MNs is also likely protective as overexpression of *Atf3* can promote MN sprouting and survival as well as retained innervation of muscle in ALS mice (Seijffers et al. 2014). *Sprr1a* is known to be an axon regeneration–induced gene (Starkey et al. 2009), suggesting a role in protective responses. Similarly, the upregulation of *Pvalb* is anticipated to be protective, as it can safeguard neurons from excitotoxicity (Van Den Bosch et al. 2002), as well as *Gal*, which also has neuroprotective properties (Elliott-Hunt et al. 2004).

Although neuromuscular junction denervation can already occur in vulnerable MN populations (Fischer et al. 2004; Valdez et al. 2012; Comley et al. 2016), the vast majority of MN somas are still present at the symptom-onset stage we examined (Rudnick et al. 2017; Chiot et al. 2020; Ribon et al. 2021). Thus, the downregulation of *Chodl*, an FF spinal MN marker, and upregulation of the S MN marker *Sv2a* could be indicative of adaptation across these two populations. It may also be an indication of subtype switching among MNs. FF MNs are highly vulnerable to ER stress and start to show patterns of denervation early in disease (Saxena et al. 2009), whereas S MNs compensate by sprouting in the mSOD1 mouse (Pun et al. 2006). Innervation of FF muscle groups by S MNs will induce muscle subtype switching toward an S type. As muscle also talks back to MNs and affects their identity (Correia et al. 2021), this may also impact FF MNs as these try to reconnect with their now modified muscle target, but this remains to be investigated. Nonetheless, some of the unique regulation in spinal MNs at the symptom onset may also represent a slight difference in proportion of MN subtypes incorporated. Future longitudinal single-cell or -nuclei RNA sequencing studies could resolve this issue.

The upregulation of *Syt7* in only spinal MNs is indicative of their need to reorganize presynaptic function, whereas the upregulation of *Lcn2*, on the other hand, appears to be a way to negatively control regeneration (Gu et al. 2024), perhaps a way to block MNs that are already handling too much stress, and DNA damage responses from regenerating, but this remains to be further investigated. Hypoglossal (CN12) MNs also showed an induction of regenerative genes, including *Gap43*, *Cd44*, and *Chl1*. Some transcripts belonging to a disease response were only regulated in the later stages in spinal MNs and were not seen in CN12, including *Adcyap1*, which is also linked to nerve regeneration (Baskozos et al. 2020). This may be a reflection that CN12 MNs are not as far along in the disease process as spinal MNs or a reflection of cell type specificity in disease response. To fully resolve this question, CN12 neurons isolated from even later stage diseased animals would need to be analyzed.

*Fgf21* was robustly and specifically upregulated in spinal MNs across disease stages. FGF21 is involved in regulating carbohydrate and lipid metabolism and maintaining energy homeostasis, and it can protect cells from apoptosis. FGF21 is induced by ER stress, mitochondrial dysfunction, and starvation. We and others have recently shown that ALS MNs display mitochondrial dysfunction early on (Hor et al. 2021; Mehta et al. 2021; Schweingruber et al. 2025), and thus, *Fgf21* upregulation may be a consequence. The upregulation of *Fgf21* in vulnerable spinal MNs may also be indicative of the increased energy demand on these cells during disease. There is a clear correlation between increased serum levels of FGF21 and metabolic disease conditions such as diabetes, mitochondrial diseases, obesity, and aging, all of which have muscle loss as a common factor (for review, see Tezze et al. 2019).

The upregulation of nuclear protein transcription regulator 1 (*Nupr1*) in CN12 and spinal MNs is indicative of the stress these cells are experiencing, as this transcriptional regulator is involved in ER stress, oxidative stress response, DNA repair, autophagy, apoptosis, and chromatin remodeling (for review, see Liu and Costa 2022). In spinal MNs, it was regulated already at the presymptomatic stage, but in CN12 neurons, it is only at the symptom-onset stage, demonstrating that these cell types follow a similar path at distinct paces, in which spinal MNs have taken the lead to destruction. Furthermore, DNA-damage inducible transcript 3 (*Ddit3* [also known as *chop*]) is an ER-stress apoptotic mediator that was upregulated in spinal MNs alone, another clear indication that these cells are furthest along a degenerative pathway. *Ddit3* mRNA was previously shown to be upregulated in spinal MNs in 90- to 120-day-old SOD1G93A mice (Perrin et al. 2005) and at 15 weeks in the SOD1G37R mouse (Lobsiger et al. 2007), as well as the protein level in end-stage sporadic ALS patient MNs (Ito et al. 2009). The earlier detection in our data, already at P56, may be a reflection of the sensitivity of Smart-seq2 and is in concordance with the very early ER stress response of fast fatigable (FF) MNs, seen prior to any visible denervation (Saxena et al. 2009). The upregulation of *Mt1* in spinal MNs across time points indicates a response to block apoptosis (Zhang et al. 2013), and *Gpnmb* also appears to be part of an inductive protective response, which is upregulated in sera from sporadic ALS patients (Tanaka et al. 2012).

Extracellular matrix remodeling is regulated by the activity of matrix metalloproteinases (MMPs) which are tightly regulated by tissue inhibitors (TIMPs). The upregulation of metallopeptidase inhibitor 1 (*Timp1*) across time points in spinal MNs and in the symptomatic stage in CN12 MNs indicates again that these two vulnerable MN groups follow similar paths of response and destruction, albeit on different timescales. *Mmp12* was also upregulated in spinal MNs, indicating ongoing ECM remodeling.

In general, we note that many gene regulations in vulnerable neurons indicate compensatory events to handle the toxicity of mutant SOD1. The majority of these appear to be beneficial, such as nerve regeneration programs, but are clearly insufficient over time. We also note that different vulnerable neuron populations share some responses to ALS, but their timing is distinct, likely owing to differences in their temporal involvement in the disease.

To further understand the strength and predictive value of the identified vulnerability signature, we compared our RNA sequencing data to that of five other published studies. We found that *Vgf* and *Tpd52l1* were identified as DEGs across all data sets independent of disease status, presymptoms, or symptom-onset stage and across SOD1G37R, SOD1G93A, and SOD1E100G mutations. A 4.8 kDa, the VGF peptide was previously described as a potential biomarker of ALS as the levels were decreased in the CSF of ALS patients and distinguished them from controls (Pasinetti et al. 2006). VGF is involved in energy expenditure, so decreased levels have been hypothesized to contribute to a hypermetabolic state in ALS. A follow-up study confirmed VGF as a biomarker for ALS and indicated that VGF CSF levels may correlate with muscle weakness in ALS patients (Zhao et al. 2008). Although *Vgf* mRNA was upregulated in our mouse data and all other mouse transcriptome studies analyzed, we found VGF protein to be decreased in ALS mouse MNs, consistent with studies on patients. The inverse correlation between RNA and protein levels indicates that there is a compensatory mechanism at play to increase VGF either by increased transcription or by changes in RNA stability.

In conclusion, we demonstrate that resilient MNs regulate few genes in response to mutant *SOD1*, likely as their baseline gene expression renders them resilient against this specific insult. The few genes that were upregulated in CN3/4 MNs have known protective properties, including *Ucn*, *Cck*, and *Postn*, and may confer resilience. We also demonstrate that CN3/4 MNs have the high baseline activity of several neuroprotective genes, including *En1*, *Gal*, *Cd63*, and *Pvalb*, that are all maintained in these neurons in ALS and that are specifically upregulated in vulnerable neurons in response to disease.

One key distinction between ALS-vulnerable spinal MNs and resilient CN3/4 neurons lies in their ability to maintain inhibitory synaptic transmission, a crucial factor in neuronal stability and protection against excitotoxicity. In spinal MNs, glycine receptor (*Glra2*) is lowly expressed compared to CN3/4 MNs but increases in ALS, possibly as a delayed compensatory mechanism to restore inhibition. Despite this, the concurrent downregulation of GABA-A receptor subunit (*Gabrg2*) suggests a continued weakening of inhibitory GABAergic input in these neurons. Additionally, CN3/4 MNs exhibit a high level of neuroprotective receptors such as GAD2, GABRD, GABRB2, GABRA1, which stabilize neuronal firing rates and prevent hyperexcitability.

We also reveal that different vulnerable MN populations share pathway activation, which indicate that cell death occurs through similar mechanisms across vulnerable MNs, but are temporally separated. The DEG and pathway analyses clearly demonstrate that vulnerable MNs activate a majority of beneficial and neuroprotective gene programs including those for nerve regeneration, reflecting their effort to reconnect. ALS-vulnerable spinal MNs exhibit increased ER stress and apoptotic signaling, with markers such as *Nupr1*, *Atf4*, and *Ddit3* promoting cell death. Additionally, *C1qb*/*C1qc* upregulation suggests that these MNs are actively marked for clearance, further driving neurodegeneration. As ALS-vulnerable MNs show *Timp1* upregulation and lower *Mmp9* levels, we speculate that this could lead to increased ECM stiffness, owing to a decreased level of ECM degradation, inflammation, and impaired axonal plasticity.

These differences in synaptic regulation, alongside the presence of neuroprotective and ECM-stabilizing factors like POSTN, suggest that CN3/4 MNs employ a distinct resilience mechanism that enables their survival in ALS, contrasting with the progressive degeneration seen in spinal MNs.

Machine learning and meta-analysis across mutant SOD1 data sets and disease time points reveal a shared transcriptional vulnerability disease code and identify *VGF*, *PENK*, *INA*, *SV2A*, and *NTS* as strong disease predictors across SOD1 mutations and species. These genes thus have potential as future biomarkers of disease and may aid in diagnosis and prognostics. In conclusion, our study reveals MN (sub)population-specific basal gene expression and temporal disease-induced regulation that together provide a basis to explain ALS selective vulnerability and resilience. Our findings also provide further support that ALS-vulnerable MNs do not simply undergo degeneration but that compensatory and neuroprotective mechanisms are at play. We reveal a number of resilience mechanisms that may provide novel therapeutic targets aimed to enhance synaptic stability and neuroprotection in vulnerable neurons.

## Methods

### Ethics statement and animal model

All procedures involving animals were approved by the Swedish ethics council and a local animal ethics committee (Paris CE5, France) and were carried out according to the code of ethics of the World Medical Association (Declaration of Helsinki). Animals were housed with a 12-h/12-h dark/light cycle under standard conditions and had access to food and water ad libitum. Adult SOD1G93A mice on a C57Bl/6J background (B6.Cg-Tg(SOD1-G93A)1Gur/J, The Jackson Laboratory strain 004435, males) were used as a model of ALS, and nontransgenic littermates served as a control. All animals were anesthetized with a lethal dose of Avertin (2,2,2-tribromoethanol in 2-methylbutanol, Sigma-Aldrich) prior to either decapitation or intracardial perfusion with phosphate buffered saline (PBS) followed by 4% paraformaldehyde (PFA) in PBS. Animals used for RNA sequencing originated from a cohort of animals located in Sweden, and all animals used for RNAscope derived from a cohort located in France.

### Tissue processing and LCM for transcriptomics

CN3/4, CN10, CN12, and spinal MNs were collected from 56- and 112-day-old male mice using LCM, as previously described (Nichterwitz et al. 2016, 2018, 2020). Twelve-micrometer coronal sections were prepared on a cryostat and placed onto PEN membrane glass slides (Zeiss). Cells were visualized by histogene staining (Arcturus/Life Technologies). Approximately 100–200 neurons were collected per sample and lysed in 0.2% Triton X-100 with 2 U/µL recombinant RNase inhibitor (Clontech). For a more detailed description, see the Supplemental Methods.

### Tissue processing for immunohistochemistry and RNAscope

The lumbar region of the spinal cords and the brains were sectioned at 30 µm. For the RNAscope experiments, we used three control and three mutant *Sod1* mice for the spinal cord at P112 and the brain regions at P56 and P112. For the spinal cord at P56, we used four mice for control and ALS.

### cDNA and sequencing library preparation

For library preparation, a modified version of the Smart-seq2 protocol (Picelli et al. 2013, 2014) was used, which is described in detail by Nichterwitz et al. (2016, 2018). Equal amounts of cDNA from samples were pooled and sequenced on the Illumina HiSeq 2500 seq platform.

### RNA-seq analysis

The RNA-seq reads were mapped simultaneously to the mm39 mouse genome assembly and the genomic sequence of human *SOD1* from the hg38 assembly using STAR (version 2.7.0e) (Dobin et al. 2013). Expression levels were determined using the rpkmforgenes.py software (https://sandberg.cmb.ki.se/rnaseq) with the Ensembl gene annotation. Samples included in the analysis had more than 15,000 detected genes (16408 ± 75 genes, mean ± SEM). Genes with at least one count in at least five samples were retained in the data set. Before differential expression analysis (DEA), samples were normalized using the calcNormFactors function from the edgeR package (Robinson et al. 2010). Following normalization, the dispersion was estimated, and the model was fitted using a quasi-likelihood (QL) approach, which provides robust error rate control for DEA. A detailed description of DEA and EA is supplied in the Supplemental Methods.

### Random forest classifier and Lasso regression classifier

To classify control and ALS samples, we implemented a random forest (RF) and Lasso regression model using the ranger and ncvreg package in R (Breheny and Huang 2011; Wright and Ziegler 2017). The input gene expression data set from Namboori et al. (2021) was preprocessed using DESeq2 normalization (v.1.34.0) (Love et al. 2014), followed by *Z*-score transformation, ensuring that each gene's expression values were centered around the mean and scaled to unit standard deviation. To address class imbalance between the ALS (n = 30) and WT (n = 85) samples, we applied downsampling using the downSample() function from the ROSE package (Lunardon et al. 2014), ensuring an equal size of classes during training. A detailed description of the model is supplied in the Supplemental Methods.

### Use of published data sets

To evaluate the purity of our samples, we compared our data to a previously published data set, in which the raw data were obtained from the NCBI Gene Expression Omnibus (GEO; accession number GSE52564) (Zhang et al. 2014) and processed as described for our own samples. Some samples used in this study were previously deposited in GEO by our laboratory with the accession numbers GSE93939 and GSE115130. We also compared out data to additional GEO data sets, including GSE40438 (Brockington et al. 2013) and GSE52118 (Kaplan et al. 2014). For the comparison of our data with other spinal MN microarray and RNA-seq data sets (Lobsiger et al. 2007; Sun et al. 2015; Namboori et al. 2021; Shadrach et al. 2021), the previously published data were preprocessed as described in the Supplemental Methods.

### RNAscope fluorescent in situ hybridization

Sections were selected by region of interest (lumbar region for the spinal cords and CN3/4, CN10 and CN12 for the brains) and subjected to RNAscope as described by Leboeuf et al. (2023) and detailed in the Supplemental Methods. The following probes (Bio-Techne) were used: Mm-Slc18a3-C3 (448771-C3), Mm-Atf3-C2 (426891-C2), Mm-Sprr1a-C2 (426871-C2), Mm-Timp1-C1 (316841), Mm-Fgf21-C1 (460931), Mm-Pvalb-C4 (421931-C4), Mm-Vgf-C2 (517421-C2), and Mm-Chl1-C1 (531051). The hybridized probes’ signals were visualized and captured on a Zeiss LSM800 Airyscan confocal microscope with a 20× objective.

### RNAscope image analysis and quantification

All tissue sections were imaged at 20× on a confocal microscope (Axio-Observer Z1/7). Scans were acquired at high resolution (4084 × 4084) with a z-step size of 0.58 µm. Maximum intensity images were first generated from merged z-focal planes using CellProfiler (v.4.2.5) (Stirling et al. 2021). The intensity of the RNAscope probes and immunohistochemistry images were quantified within the masked *VAChT*/ChAT^+^ cells using an automated CellProfiler pipeline, as described in detail in the Supplemental Methods.

### Spinal cord immunohistochemistry

Sections were quickly washed in PBS and incubated in 5% donkey serum, 0.3% Triton X-100 in PBS for 1 h at RT. Sections were then incubated overnight with primary antibodies diluted in 5% donkey serum, 0.3% Triton X-100 in PBS at 4°C and washed and further incubated with secondary antibodies for 1 h at RT. Primary antibodies included rabbit anti-VGF (LSBio LS-C352987, 1:100) and goat anti-VAChT (Millipore ABN100, 1:500). Controls without primary antibodies were included. Images were acquired on a Zeiss LSM800 Airyscan confocal microscope with a 20× objective.

## Data access

All LCM-seq RNA-seq data generated in this study have been submitted to the NCBI Gene Expression Omnibus (GEO; https://www.ncbi.nlm.nih.gov/geo/) under accession number GSE244538.

## Supplemental Material

Supplement 1

Supplement 2

Supplement 3

Supplement 4

Supplement 5

Supplement 6
