# Supplementary material for: Transcriptional modulation unique to vulnerable motor neurons predicts ALS across species and SOD1 mutations
Source: Genome Res. 2025 Sep;35(9):1975–91. doi: 10.1101/gr.279501.124 (PMC12400948; doi:10.1101/gr.279501.124)
Supplement: Supplement 1 [file Supplemental_material_Mei_et_al.pdf]

Supporting online material for:

Transcriptional modulation unique to vulnerable motor neurons predicts ALS across species and SOD1 mutations

Irene Mei[1], Susanne Nichterwitz[1,3], Melanie Leboeuf[1,2], Jik Nijssen[2,3], Isadora Lenoel[4], Dirk Repsilber[5], Christian S. Lobsiger[4], Eva Hedlund[1,2,3]

Corresponding author: eva.hedlund@dbb.su.se

This document includes Supplemental Methods and Supplemental Figures S1-9.

Supplemental Methods

Tissue processing and laser capture microdissection for transcriptomics

For the dissection and processing of tissues for LCM-seq, all equipment, surfaces, and tools were carefully cleaned with RNaseZap (Ambion/Life Technologies) and wiped with distilled water or 70% ethanol. Brain and spinal cord tissues were dissected from 56 and 112-day old males and immediately snap-frozen in 2-Methylbutane (Sigma-Aldrich) on dry ice, followed by storage at -80°C until further processing. After equilibration of the tissue to approximately -20°C, 12 µm coronal sections were prepared on a cryostat and placed onto PEN membrane glass slides (Zeiss). Spinal cords were embedded in OCT (Dako) prior to sectioning. Slides were stored at -80°C until further processing.

For laser capture microdissection (LCM), cells were visualized with a quick histological staining (Histogene, Arcturus/Life Technologies) followed by dehydration in a series of ethanol solutions (75%, 95%, and 99.7%, Solveco). We made the choice of including CN10 vagus neurons as a resilient motor nucleus, for the following reasons; i) CN10 neurons represent a relatively ALS-resistant MN population with relatively long axons. ii) We wanted to study a classically visceral nucleus. iii) Finally, CN10 neurons are located within a readily identifiable nucleus histologically as well as size wise in the mouse. LCM was performed on a Leica LMD 7000 system at 40x magnification and cutting outlines were drawn in close proximity to individual cells to minimize contamination by surrounding tissue.

Approximately 100-200 cells with an area of >200 µm^2 (150 µm^2 for vagus motor neurons) and a visible nucleus with nucleolus were collected per sample into the dry cap of a PCR tube (Biozym Scientific). After the addition of 5 µl lysis buffer (0.2% Triton X-100, with 2 U/µl recombinant RNase inhibitor, Clontech) to the cap, samples were mixed by pipetting up and

down, spun down in a tabletop centrifuge and snap frozen on dry ice. Samples were stored at -80°C until library preparation.

Tissue processing for immunohistochemistry and RNAscope

Male and female mice were transcardially perfused with PBS followed by 4% PFA in PBS at P56 or P112. Brains and the lumbar region of the spinal cord was dissected, post-fixed for 3 h in 4% PFA at 4°C, and cryoprotected in PBS 30% sucrose for 48 h at 4°C. The lumbar region of the spinal cords and the brains were sectioned at 30 µm thick using Thermo Scientific Sliding Microtome Microm HM 430. For the RNAscope experiments we used three control and three mutant SOD1 mice for spinal cord at P112 and brains regions at P56 and P112. For the spinal cord at P56 we used four mice for control and ALS.

cDNA and sequencing library preparation

For RNA-seq experiments, all reagents were of molecular biology/PCR grade if available. Only nuclease-free water (H_2O, LifeTechnologies) and tubes that were certified nuclease-free were used. All workbenches and equipment were cleaned with RNaseZAP (Ambion/Life Technologies) and additionally with DNAoff (Takara) for library preparations.

For library preparation, a modified version of the Smart-seq2 protocol (Picelli et al. 2014b, 2013) was used, which is described in detail in (Nichterwitz et al. 2018, 2016). All heat incubation steps and PCR cycles were carried out in a BioRad T100 Thermal Cycler. After reverse transcription, cDNA was amplified for 18 cycles, followed by purification with magnetic beads (GE Healthcare). cDNA concentration and library quality were assessed using an Agilent 2100 Bioanalyzer (High Sensitivity DNA kit). Three samples with poor cDNA library

quality were excluded from analysis (low cDNA concentration with no apparent peaks). One ng of cDNA (as determined with the Bioanalyzer, 100-9000 bp range) was used as input for the tagmentation reaction, which was carried out with 0.4 -1 µl of in house Tn5 (Picelli et al. 2014a). Ligation of sequencing indices (Nextera XT Sequencing Index Kit, Illumina) and 10 cycles of enrichment PCR were performed with Kapa HiFi polymerase. Final sequencing libraries were purified with magnetic beads and the concentration of each sample was determined on a Qubit fluorometer (ThermoFisher) using the dsDNA high sensitivity kit (Life Technologies). Samples were pooled and sequenced on the Illumina HiSeq 2500 seq platform.

RNA-seq analysis

Differential expression analysis was performed using the R-package edgeR (v3.36.0 Robinson et. al. 2010). A quasi-likelihood negative binomial generalized log-linear model was fitted to the data before empirical Bayes moderated t-statistics were calculated and multiple testing correction (Benjamin-Hochberg) was performed. The threshold false discovery rate (FDR) < 0.05 was used to determine if a transcript was differentially expressed. No minimum fold change was applied to identify differentially expressed genes, but the smallest fold change in gene expression was 0.94 of log2FC. For Supplemental Fig. S2D, an ANOVA was implemented with one factor (Cell type_ Age) which was significant. We then ran post-hoc analyses to assess pairwise differences and *t*-test results with Bonferroni correction are reported.

Pairwise sample correlations were computed using Pearson's method on variance-stabilized (VST) expression values, and visualized as a heatmap to assess clustering by cell type (see Fig. , Supplemental Fig. S2D).

Enrichment analysis

The Enrichment analysis methods used in this study belong to three different categories: OVerlap Analysis (OVA), Per-Gene score Analysis (PGA) and Network Enrichment Analysis (NEA) methods. As methods, we selected EASE (OVA), FGSEA (Korotkevich et al. 2021, PGA) and ANUBIX (Castresana-Aguirre M et al. 2020, NEA). NEA methods stand out from other approaches due to their consideration of the interconnections among DEGs within the context of functional gene sets in a network framework (Buzzao et al. 2024). All methods were run in an R (v4.1.1) version-controlled conda environment using the original packages, EASE and ANUBIX were run as in a recent benchmarking study for gene set EA methods (Buzzao et al. 2024). EASE and ANUBIX were performed on differentially expressed genes with an FDR below 0.1. FunCoup (Alexeyenko and Sonnhammer, 2009; https://funcoup.org/search/) was used as a network with a default confidence link score of 0.8 and 5 max neighbors in order to obtain the network of links that exist between vulnerable genes within detrimental pathways. Gene Set Variation Analysis (Hänzelmann S. et al. 2013, GSVA, v.1.50.1) was performed to assess pathway enrichment. GSVA was used on log-transformed counts per million (CPM) values calculated from the expression data. A GSVA parameter object was then created with the 'gsvaParam' function, using log-transformed CPM values, list of GO-BP gene sets (MSigDB), kcdf=Gaussian and maxDiff=False. The 'gsva' function was subsequently applied to compute enrichment scores for each sample.

Use of published datasets

To evaluate the purity of our LCM collected samples, we compared our data to a previously published dataset obtained from GEO, accession number GSE52564 (Zhang et al. 2014), which was processed as described for our samples. For analysis of oculomotor versus spinal motor neuron enriched transcripts across species we compared with our published RNA sequencing

data set from Allodi et al (GSE93939) and Nizzardo et al (GSE115130), with Brockington et al. 2013 (GSE40438) and Kaplan et al. 2014 (GSE52118). For the comparison of our data with other spinal motor neuron microarray and RNA-seq data, the previously published data were preprocessed as described below. The microarray data (Lobsiger et al. 2007) were analyzed after rma() normalization and quality control for both moe430A and B arrays. Shadrach et al. 2021 and Sun et al. 2015 were mapped to the mm39 assembly, using STAR (version 2.7.0e). Genes with a minimum count of 4 and 3, respectively, within all samples were retained. Namboori et al 2021, was mapped to the hg38 assembly and processed retaining all MN cells (n=115 cells) with co-expression of *Slc18a3* and *Isl1* of at least 5 counts and nUMI >= 1000, nGene >= 300 and mitoRatio<= 0.5). This subset of cells was used for the Random Forest and Lasso regression classification analysis. Differential analysis was carried out using Wald test in DESeq2 and parameters set as follows: sfType = "poscounts", minReplicatesForReplace = Inf, useT = TRUE and minmu = 1e-06. We chose to use DESeq2 for the differential expression analysis for the Namboori dataset, to ensure comparability and consistency, as this methodology was used in the original paper, with the reported results (DESeq(dds, test = "Wald", sfType = "poscounts", minReplicatesForReplace = Inf, useT = TRUE, minmu = 1e-06)). To our knowledge, there is no dedicated function specifically designed for single-cell differential expression analysis in edgeR that provides comparable optimization possibilities to DESeq2 without resorting to pseudo-bulking.

Random forest classifier

To identify genes predictive of ALS versus WT motor neurons, we used a Random Forest (RF) classifier with permutation-based variable importance and leave-one-out cross-validation (LOOCV). Input features consisted of gene expression values scaled by z-score normalization

across samples. We trained the RF model using the ranger algorithm with hyperparameter tuning over a grid of mtry, min.node.size, and fixed splitrule = "gini". To account for class imbalance (ALS vs WT), we performed repeated downsampling (10 iterations), training a random forest model on each balanced dataset. For each model, we computed permutation-based variable importance. Genes were ranked by their average importance across the 10 runs, and the top 30 features were selected for further analysis. We also recorded how frequently each gene appeared among the top-ranked features. Model performance was assessed via LOOCV on the full dataset (n = 115), and metrics including sensitivity, specificity, PPV, NPV, accuracy, and AUC were recorded. To assess statistical significance, we repeated the entire LOOCV pipeline with 500 random permutations of the class labels and computed permutation-based p-values for each performance metric.

Lasso regression

To identify genes predictive of ALS versus control samples, we performed LASSO (Least Absolute Shrinkage and Selection Operator) logistic regression using the ncvreg R package. Gene expression data were first z-score standardized across samples. We limited our analysis to a subset of genes that were pre-selected based on differential expression results obtained from an independent dataset (upregulated DEGs in spinal cord at P112). Model fitting was conducted using cross-validated penalized logistic regression with LASSO regularization. To account for variability in gene selection due to model stochasticity, we repeated model training 500 times, each time recording the coefficients of selected features. The importance score for each gene was calculated as the mean coefficient across these repetitions. Genes with a non-zero mean coefficient (positive or negative) were considered consistently selected features. To assess classifier performance, we implemented leave-one-out cross-validation (LOOCV) and computed standard metrics including sensitivity, specificity, positive predictive

value (PPV), negative predictive value (NPV), and area under the ROC curve (AUC). To evaluate statistical significance of model performance, we conducted 500 permutations of class labels, repeating the LOOCV and recalculating performance metrics to derive permutation p-values. Importantly, LASSO and Random Forest are fundamentally different models, and they capture different gene expression patterns: LASSO selects a small set of genes that individually show strong, consistent differences between ALS and control. Random Forest, on the other hand, can detect more subtle patterns that depend on combinations of genes rather than any single one. Therefore, the two approaches can highlight complementary sets of features.

Tissue processing for immunohistochemistry and RNAscope

Male and female mice were transcardially perfused with PBS followed by 4% PFA in PBS at P56 or P112. Brains and the lumbar region of the spinal cord was from perfused animals were dissected, post-fixed for 3 h in 4% PFA at 4°C, and cryoprotected in PBS 30% sucrose for 48 h at 4°C. The lumbar region of the spinal cords and the brains were sectioned at 30 µm using Thermo Scientific Sliding Microtome Microm HM 430.

RNAscope fluorescent *in situ* hybridization

Selected tissue sections were washed in PBS, incubated with RNAscope hydrogen peroxide solution from Advanced Cell Diagnostics (ACD) for 10 min at room temperature (RT), rinsed in PBS 0.1% Tween 20 at RT, collected on Super Frost plus microscope slides (Thermo Scientific), dried at RT for 1 h, rapidly immersed in ultrapure water, dried again at RT for 1 h, heated for 1 h at 60°C, and dried at RT overnight. The next day, sections were immersed in ultrapure water, rapidly dehydrated with 100% ethanol, incubated at 100°C for 15 min in RNAscope 1X Target Retrieval Reagent (ACD), washed in ultrapure water, and dehydrated

with 100% ethanol for 3 min at RT. Protease treatment was carried out using RNAscope Protease Plus solution (ACD) for 30 min at 40°C in a HybEZ oven (ACD). Sections were then washed in PBS before *in situ* hybridization using the RNAscope Multiplex Fluorescent V2 Assay (ACD). Probes were hybridized for 2 h at 40°C in a HybEZ oven (ACD), followed by incubation with signal amplification reagents according to the manufacturer's instructions.

RNA scope Image analysis and quantification

All tissue sections from ages P56 and P112 were imaged at 20X on a confocal microscope (Axio- Observer Z1/7). Scans were acquired at high resolution (4084 × 4084) with a z-step size of 0.58 µm. Maximum intensity images were firstly generated from merged z-focal planes using CellProfiler (Stirling et al. 2021, v.4.2.5). Intensity of RNAscope probes and immunohistochemistry images were quantified within the masked *Vacht*/ChAT+ cells using an automated CellProfiler pipeline. Each cellular specimen exhibiting an intensity of *Vacht*/ChAT+ signal higher than 0.01 was subjected to quantification. Each image, after being z-projected using Cellprofiler, was processed following the steps below: The images were imported as grayscale. The main approach started applying median filtering to the *Vacht* channel of the images using the 'MedianFilter' module**.** We calculated illumination correction with the 'CorrectIlluminationCalculate' module and then applied it using the CorrectIlluminationApply module for all the channels of the image (see Supplemental Figure 4A). This step allowed correction for possible illumination unbalance. We then enhanced the features signal of the *Vacht* channel to identify MNs better. We then used the 'IdentifyPrimaryObjects' module to identify the MNs and mask them, employing per-object thresholding and manual adjustments to the smoothing filter size and maximal suppression distance to optimize segmentation. Following this, we measured the intensity of the masked

MNs for all the other channel probes we were interested in with the ‘MeasureObjectIntensity’ module and assessed their size and shape using the ‘MeasureObjectSizeShape’ module. We placed outlines on the images using the ‘OverlayOutlines’ module and saved the processed images with the ‘SaveImages’ module. We repeated this semi-automatic pipeline for all the images of all the conditions and ages. Statistical testing was performed on cells with a mean raw intensity > 0.8 in both conditions. A one-tailed permutation test (100,000 permutations) was used to assess significance, and nominal p-values are reported.

APA quantification

The MAAPER software (v. 1.1.1) was used to map sequencing reads to known polyadenylation (PA) sites, as specified in the PolyA DB v3 database for the mm39 genome (Li et al. 2021; Wang et al. 2018). To detect genes exhibiting significant alterations in the length of their 3′-most exon, we applied the REDu metric provided by MAAPER. REDu quantifies the relative expression levels of the two most differentially expressed isoforms in the 3′-most exon. Positive REDu values signify transcript lengthening, while negative values indicate shortening events. To control for multiple testing, we applied the Benjamini-Hochberg (BH) correction to the REDu p-values, obtaining FDR-adjusted p-values (FDR_pval). Genes with FDR_pval < 0.05 were considered significantly altered in APA.

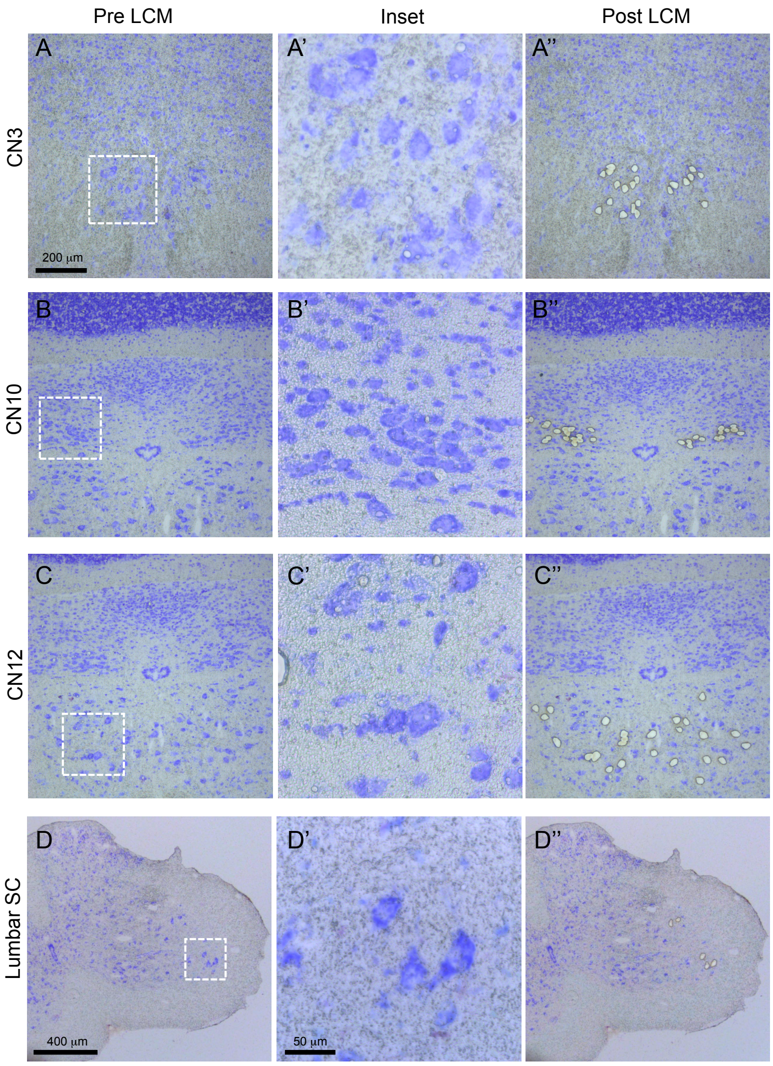

Supplemental Figure S1. Laser capture microdissection (LCM) of neurons from brain motor nuclei and spinal cord. (**A**-**D**) Representative brightfield images showing motor neuron populations before and after LCM in the oculomotor/trochlear nucleus (CN3/4) (**A**), dorsal motor nucleus of the vagus nerve (CN10) (**B**), hypoglossal nucleus (CN12) (**C**), and lumbar spinal cord (SC) (**D**). Motor neurons were stained with Histogene (Arcturus) prior to dissection to enable precise visualization. (**A**'-**D'**) Close-up images of the boxed regions in A-D highlight motor neuron morphology prior to laser capture. (**A''**-**D''**) Images after LCM showing successful motor neuron removal, with the remaining tissue demonstrating cleanly excised neuronal profiles. Scale bars: A = 200 µm (applies to A, A'', B, B'', C, C''); D = 400 µm (for D and D''); D' = 50 µm (applies to A', B', C', D').

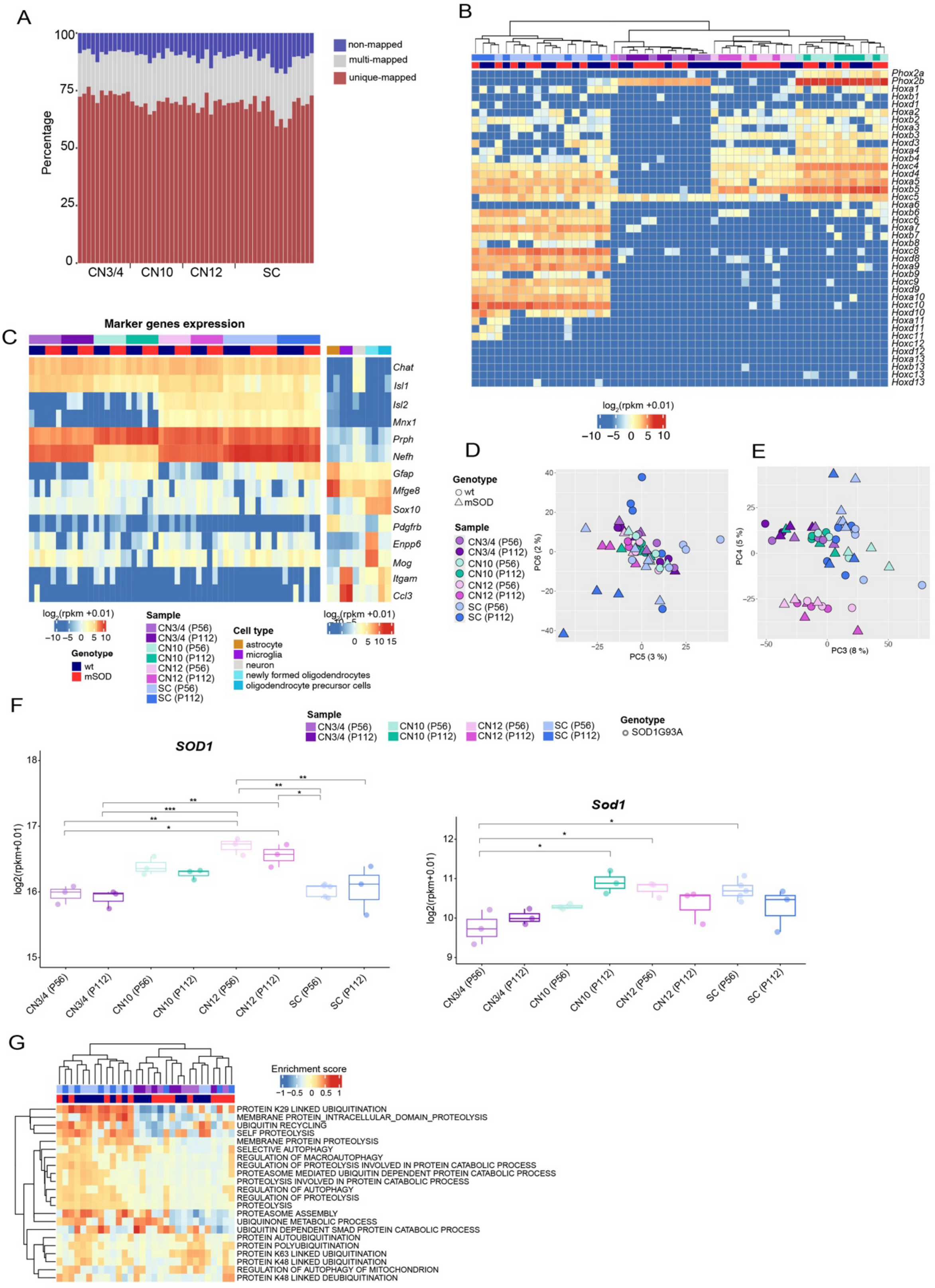

Supplemental Figure S2. Positional identity and neuronal enrichment validation in motor neuron subpopulations. (**A**) Mapping statistics of RNA-seq samples (n=54) mapped to the mouse genome (mm39) with an added human *SOD1* sequence. It shows the percentage of uniquely mapped, multi-mapped, and non-mapped reads per motor neuron subtype. Only uniquely mapped reads were used

in downstream analyses. (**B**) Heatmap of Hox gene expression profiles, evaluating the positional identity of motor neuron populations along the rostrocaudal axis. Hox gene expression patterns confirm expected regional identity of neurons from CN3/4, CN10, CN12, and spinal cord (SC). (**C**) Heatmap of neuronal marker gene expression, confirming an enrichment of motor neurons in the dataset. LCMseq samples were compared to a previously published RNA-seq data set of neurons (black), astrocytes (orange), oligodendrocyte precursor cells (OPCs, light turquoise), newly formed oligodendrocytes (NFO, medium turquoise) and myelinating oligodendrocytes (MO, turquoise) to evaluate the purity of our neuronal samples (GEO accession number GSE52564). (**D-E**) Principal component analysis (PCA) of additional PCs (PC3-6) did not show further clustering of disease versus control. (**F**) Boxplots visualizing RNA levels of the human SOD1 transgene (top) and endogenous mouse *Sod1* (bottom) in transgenic animals. Data indicate robust transgene expression in ALS mice and consistent levels of endogenous *Sod1* across cell types. Statistical tests: one-way ANOVA with Bonferroni's multiple comparison test (*P ≤ 0.05; **P ≤ 0.01; ***P ≤ 0.001; ****P ≤ 0.0001). (**G**) Heatmap of Gene Ontology (GO) enrichment scores from Gene Set Variation Analysis (GSVA), assessing pathways related to ubiquitin-proteasome function, protein degradation, and autophagy-related processes in CN3/4 and spinal motor neurons of control and SOD1G93A mice.

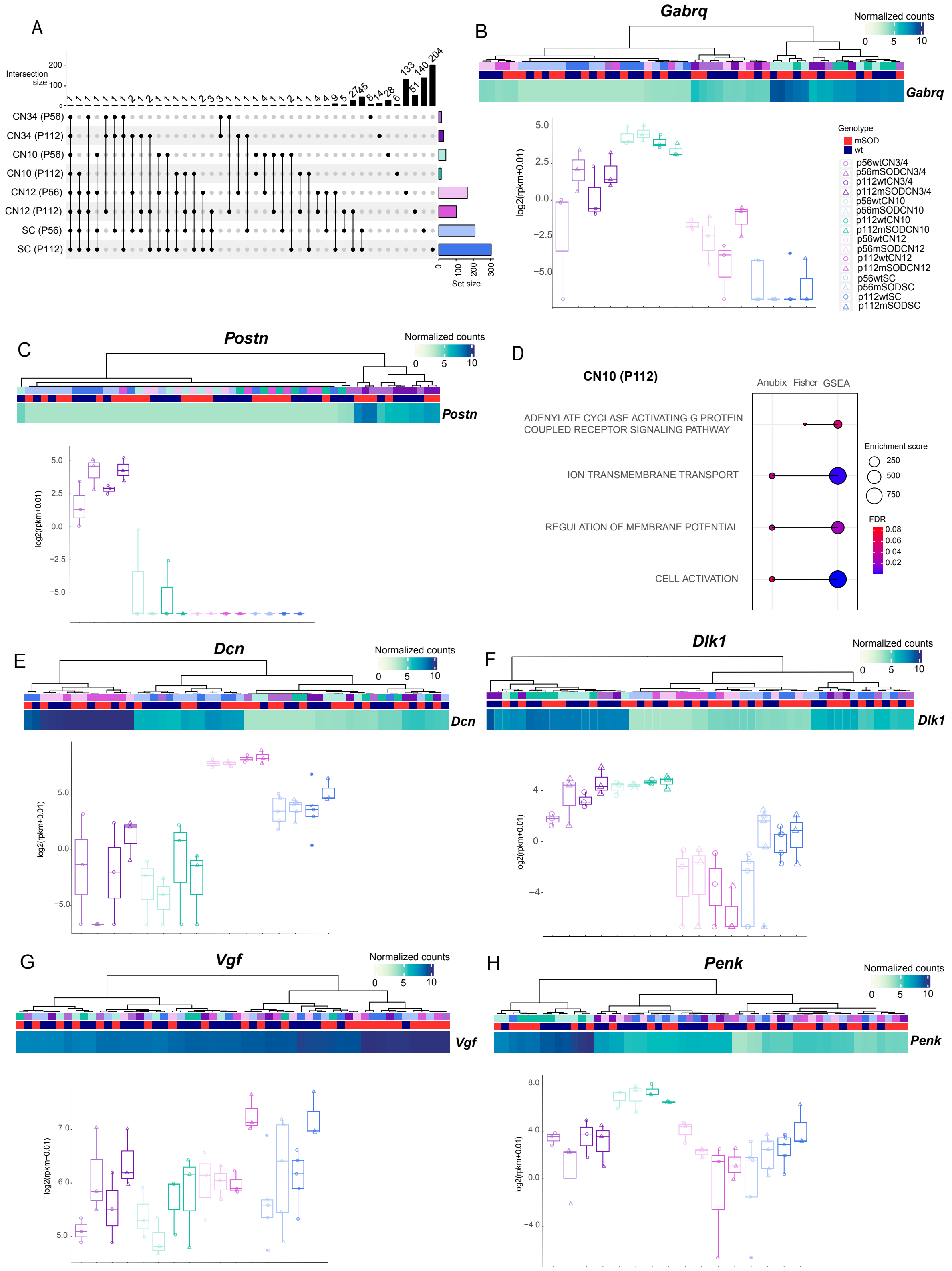

Supplemental Figure S3. Differential gene expression of selected genes and Gene Ontology (GO) enrichment analysis for CN10. (**A**) Upset plot showing the overlap of differentially expressed genes (DEGs) across all motor neuron subtypes and ages. This visualization highlights shared and unique DEGs across CN3/4, CN10, CN12, and SC at P56 and P112, showing common pathways between resistant and vulnerable populations. (**B-C, E-H**) Expression patterns of selected genes in different

motor neuron subtypes and ages. Heatmaps (top panels) display normalized counts of each gene across all samples, while boxplots (bottom panels) show log2-transformed RPKM expression levels, highlighting differences between genotypes (SOD1G93A vs. WT) and cell types; (**B**) *Gabrq*, (**C**) *Postn*. (**E**) *Dcn,* (**F**) *Dlk1*, (**G**) *Vgf*, (**H**) *Penk*. (**D**) GO term dot plot representing enriched biological processes in CN10 at P112. The Gene Ontology (GO) enrichment analysis was performed using three complementary generations of enrichment methods: Fisher's exact test, functional gene set enrichment analysis (FGSEA), and Anubix. Enrichment scores correspond to the amount of functional genes that the method shows being related for the enrichment term. FDR threshold < 0.1.

A

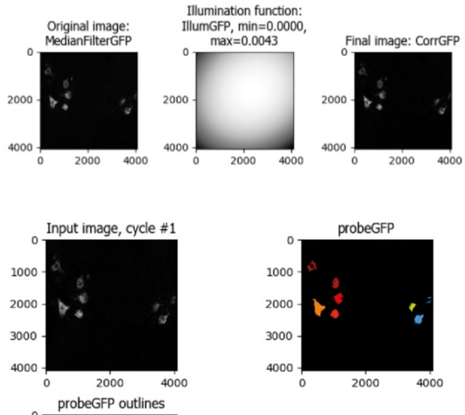

Number of accepted objects	9
10th pctile diameter	73.3 pixels
Median diameter	242.8 pixels
90th pctile diameter	355.4 pixels
Area covered by objects	2.4%
Thresholding filter size	1.0
Threshold	0.0436

B

		SC				CN12				CN10				CN34			
		P56		P112		P56		P112		P56		P112		P56		P112	
		WT	ALS	WT	ALS	WT	ALS	WT	ALS	WT	ALS	WT	ALS	WT	ALS	WT	ALS
Probes present in the same mix	*Atf3*	407	281	340	136												
	Timp1			340	136												
	Vgf			1031	704												
	Sprr1a			321	218												
	Pvalb	606	523														
	Fgf21	592	508	434	258												
	Atf3					488	300	308	289					221	198	262	192
Probes present in the same mix	*Chl1*							530	508			377	206			376	319
	Sprr1a							530	508							376	319
Probes present in the same mix	*Timp1*							323	297							303	132
	Pvalb					579	238							278	131		
	Fgf21					488	300							221	198		

Supplemental Figure S4. Image processing and quantification workflow for RNAscope analysis. (A) Pre-processing and segmentation workflow for RNAscope images using CellProfiler. The RNAscope probe used for VAChT/ChAT detection was GFP-labeled. The top row displays the pre-processing steps: the original image after applying a median filter (left), the illumination correction function used to normalize the image (center), and the final corrected image (right). The bottom row shows the segmentation workflow: the input image from the first analysis cycle (left), the segmented probeGFP objects highlighted in different colours (center), and the final outlines of the detected objects overlaid on the corrected image (right). The table on the bottom right provides example statistics, including the number of detected objects (n = 9), object size metrics (10th, median, and 90th percentile diameters) and the area covered by objects (2.4%). (**B**) Table showing the number of motor neurons that were quantified for the RNAscope probes used.

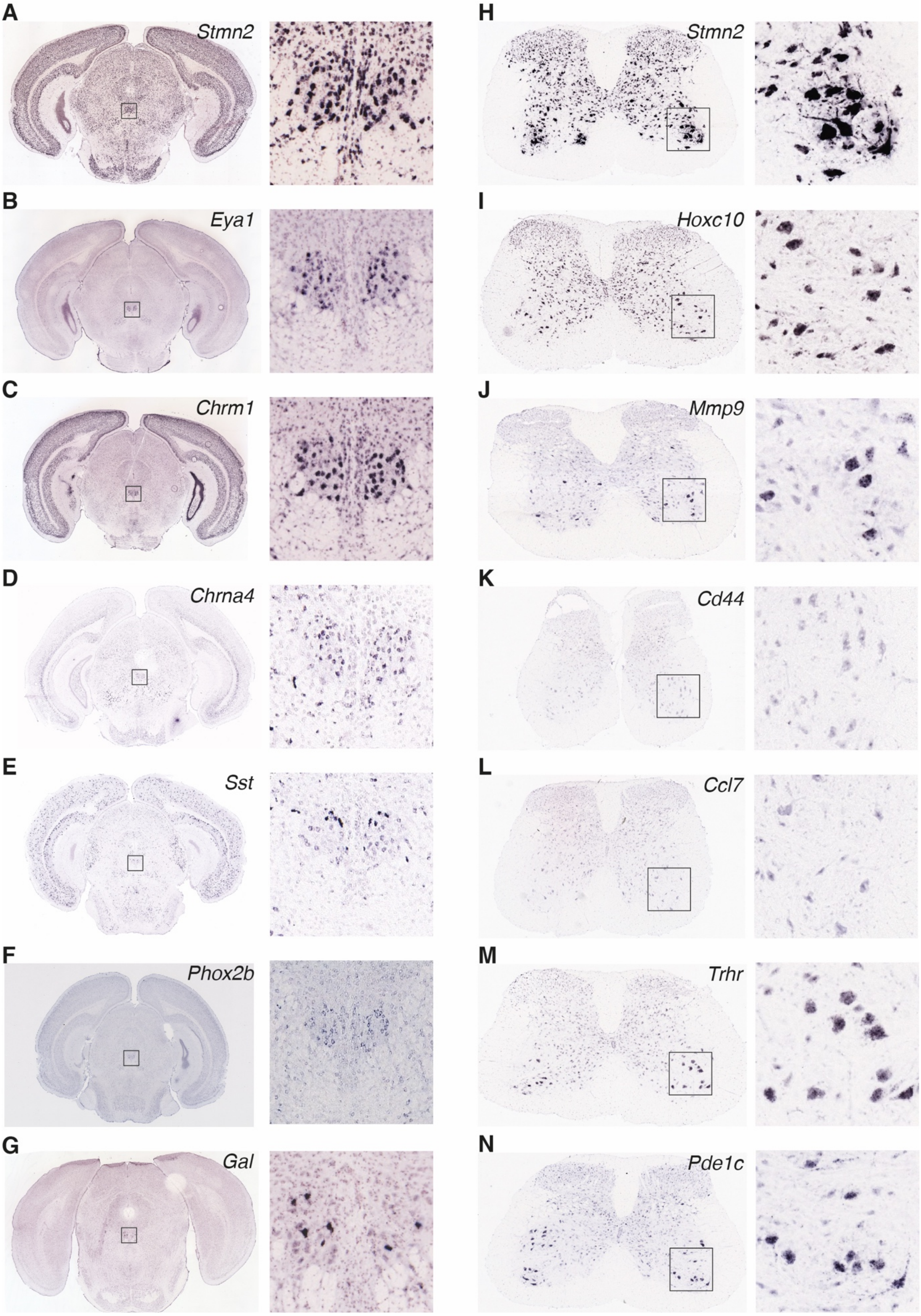

Supplemental Figure S5. Validating the expression of selected DEGs in motor neurons.
(A-G) We confirmed the mRNA localization of *Stmn2, Eya1, Chrm1, Chrna4, Sst, Phox2b* and *Gal* in CN3/4 (enlarged on the right panel) of P56 mouse brains. **(H-N)** We confirmed the mRNA localization

of *Stmn2, Hoxc10, Mmp9, Cd44, Ccl7, Trhr* and *Pde1c* in the spinal motoneurons (enlarged on the right panel) of P56 mice. (© 2004 Allen Institute for Brain Science. Allen Mouse Brain Atlas and Allen Mouse Spinal Cord Atlas. Available at: http://mouse.brain-map.org/ and http://mousespinal.brain-map.org)

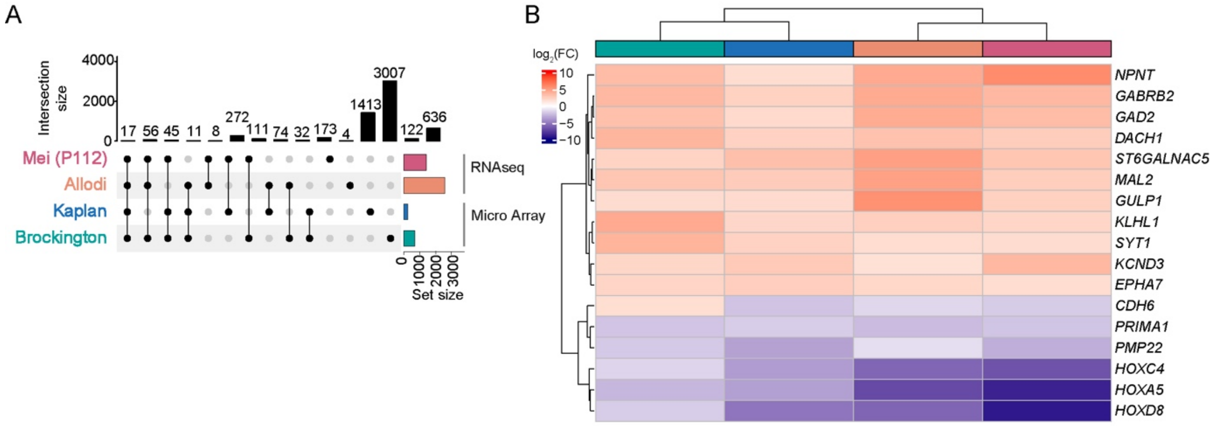

Supplemental Figure S6. Differentially expressed genes (DEGs) in wild-type baseline datasets between ocular motor neuron (OMN; CN3/4) and spinal motor neuron (SC). (**A**) Upset plot of DEGs across multiple datasets comparing OMN vs. SC in SCP112 (Mei et al.), Allodi et al. (2019), Kaplan et al. (2014), and Brockington et al. (2013). Mei et al. and Allodi et al. (2019) are RNAseq dataset and Kaplan et al. (2014) and Brockington et al. (2013) microarray datasets. (**B**) Heatmap of DEGs that were shared across datasets, clustered by expression profile similarity. Genes are color-coded based on expression intensity (log_2(FC) between OMN and SC), with upregulated genes in warm tones (orange-red) and downregulated genes in cool tones (blue-purple).

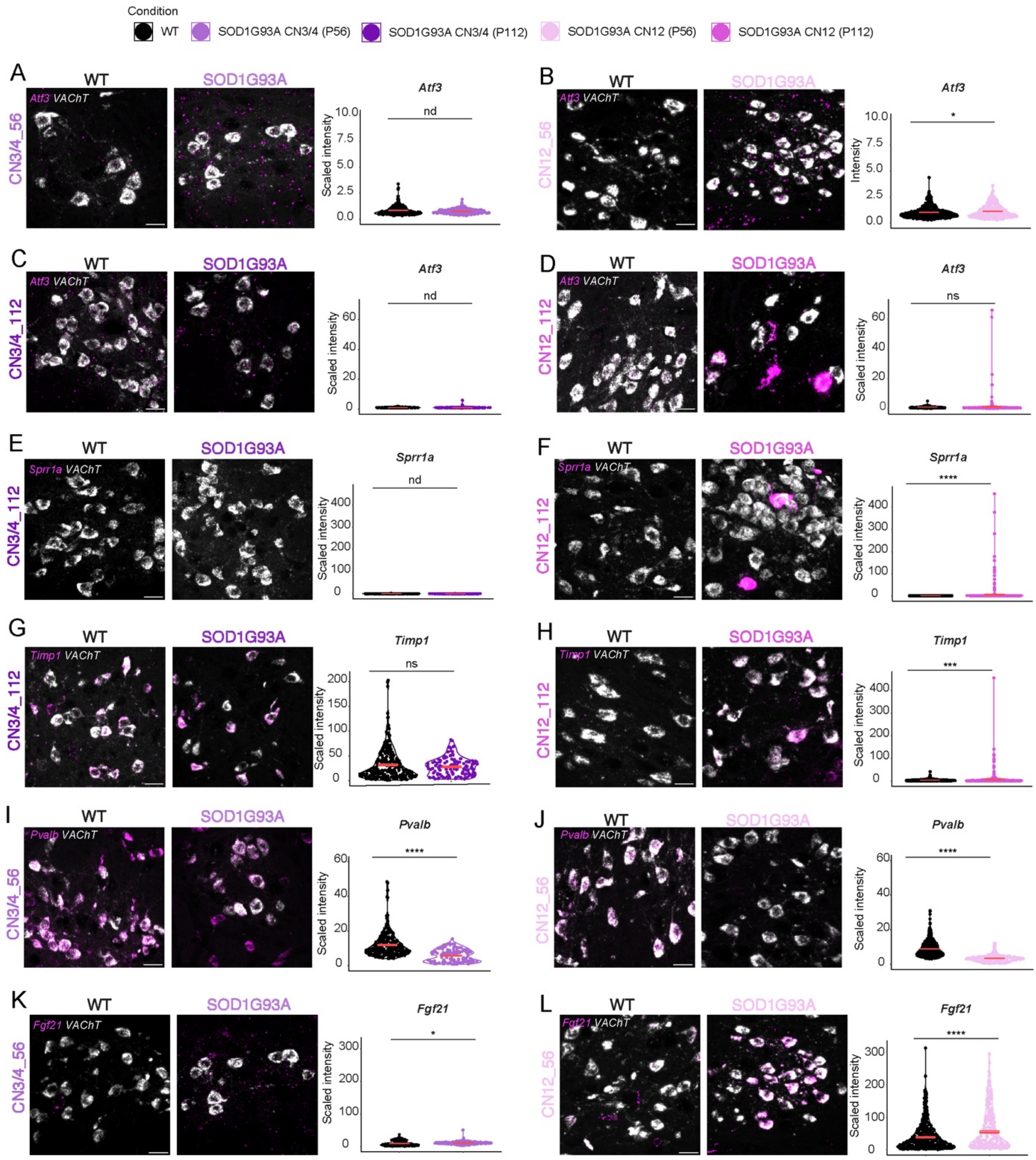

Supplemental Figure S7. RNAscope analysis in CN3/4 and CN12 motor neurons at different ages. (**A-L**) Representative RNAscope images and quantifications of transcript scaled intensity (raw intensity multiply by a scaling factor of 1000) across CN3/4 and CN12 motor neurons in WT and SOD1G93A mice. Images show fluorescent in situ hybridization signals for selected genes, with corresponding quantification of fluorescence intensity per motor neuron. (**A**) Representative RNAscope image with quantification of signal intensity of *Atf3* in CN3/4 at P56 (n for WT= 221; n for SOD1G93A= 198), (**B**) in CN12 at P56 (n for WT= 488; n for SOD1G93A= 300), (**C**) in CN3/4 at P112 (n for WT= 262; n for SOD1G93A= 192), (**D**) in CN12 at P112 (n for WT= 308; n for SOD1G93A= 289). (**E**) *Sprr1a* in CN3/4 at P112 (n for WT= 376; n for SOD1G93A= 319), (**F**) and in CN12 at P112 (n for WT= 530; n for SOD1G93A= 508). (**G**) *Timp1* in CN3/4 at P112 (n for WT= 303; n for SOD1G93A= 132), (**H**) and in CN12 at P112 (n for WT= 323; n for SOD1G93A= 297). (**I**) *Pvalb* in CN3/4 at P56 (n for WT= 278; n for SOD1G93A= 131). (**J**) in CN12 at P56 (n for WT= 579; n for SOD1G93A= 238). (**K**) *Fgf21* in CN3/4 at P56 (n for WT= 221; n

for SOD1G93A= 198), (**L**) and in CN12 at P56 (n for WT= 488; n for SOD1G93A= 300). (A-L) Scale bars: 30 µm. Significance of the quantification performed using one tail mean permutation test; nd: not detectable (mean raw intensity < 0.8 in both genotypes), ns: P> 0.05, *: P ≤ 0.05, **: P ≤ 0.01, ***: P ≤ 0.001, **: P ≤ 0.0001)

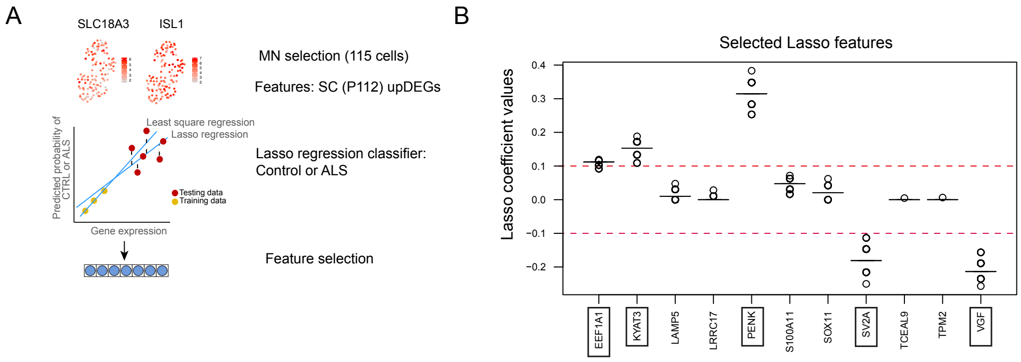

Supplemental Figure S8. Feature selection using LASSO regression to classify ALS vs Control samples. (**A**) Schematic of the Lasso classification workflow. Motor neurons (MNs) were selected based on expression of MN markers (*SLC18A3* and *ISL1*). Gene expression data from Namboori et al. (n=115 cells) were used as samples, restricted to genes upregulated in ALS (SC P112 upDEGs). A LASSO logistic regression model was trained to classify samples as ALS or Control using these features. The LASSO regression line fits the data more conservatively than least squares regression, enforcing sparsity in selected features.(**B**) Boxplots of gene coefficients from LASSO regression across 500 repeated fits. Displayed genes are those with a non-zero average coefficient, indicating consistent selection across repetitions.

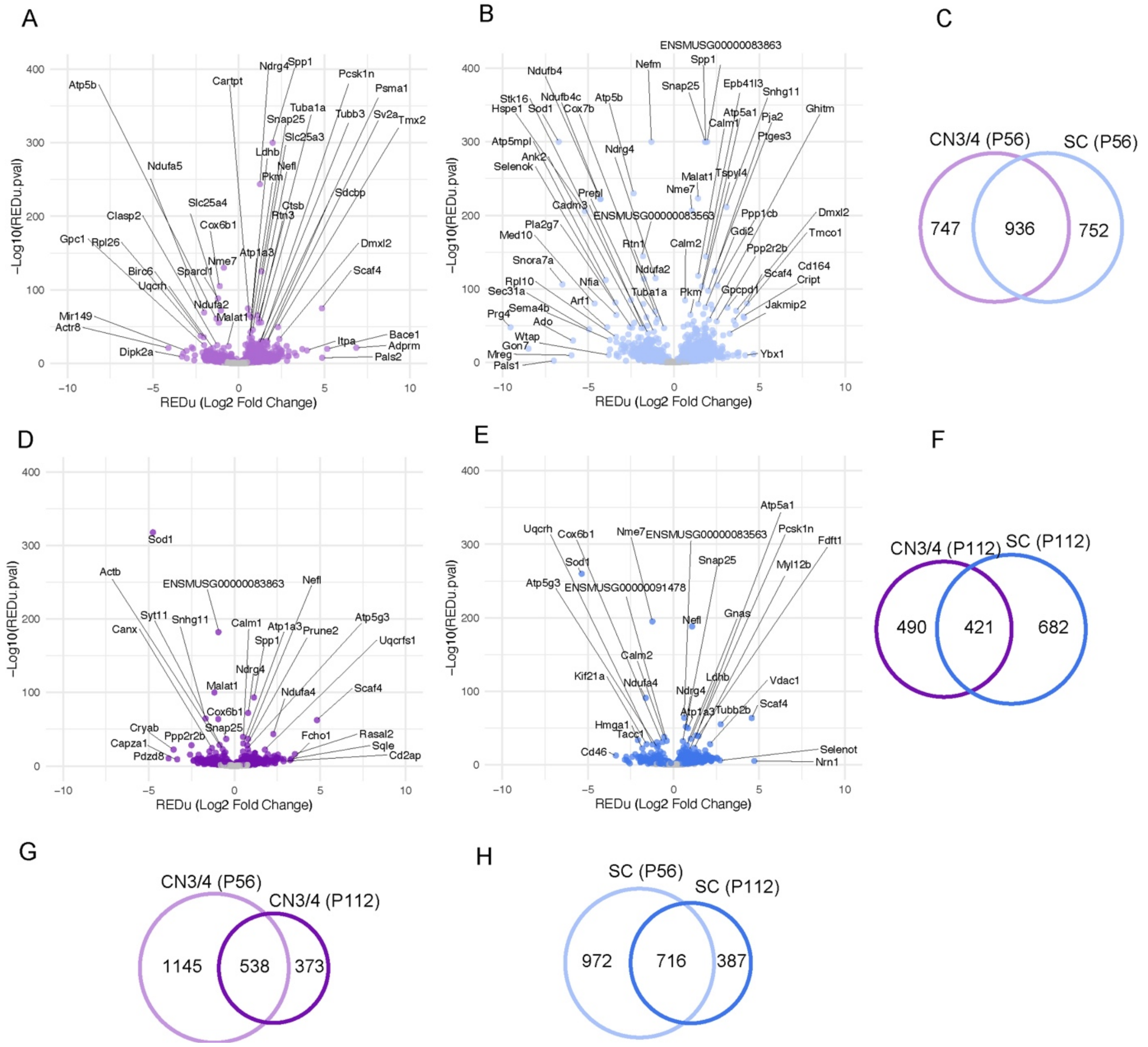

Supplemental Figure S9. Alternative Polyadenylation analysis in CN34 and SC.

(A, B, D, E) Volcano plots showing differential alternative polyadenylation (APA) site usage as assessed by the REDu metric. The x-axis represents REDu (log2 fold change), while the y-axis shows the -log10 p-value. Positive REDu values indicate transcript lengthening, and negative values indicate shortening. Genes with the most significant REDu changes are labelled. (**A**) Comparison of CN3/4 at P56. (**B**) Comparison of SC at P56. (**D**) Comparison of CN3/4 at P112. (**E**) Comparison of SC at P112. **(C, F, G, H)** Venn diagrams illustrating the overlap of genes with significant APA changes (FDR < 0.05) between different conditions. (**C**) CN3/4 at P56 vs. SC at P56. (**F**) CN3/4 at P112 vs. SC at P112. (**G**) CN3/4 at P56 vs. CN3/4 at P112. (**H**) SC at P56 vs. SC at P112.
